# Gut Bacteria of Water Monitor Lizard (*Varanus salvator*) Are a Potential Source of Antibacterial Compound(s)

**DOI:** 10.3390/antibiotics8040164

**Published:** 2019-09-24

**Authors:** Noor Akbar, Ruqaiyyah Siddiqui, K Sagathevan, Mazhar Iqbal, Naveed Ahmed Khan

**Affiliations:** 1 Department of Biological Sciences, School of Science and Technology, Sunway University, Bandar Sunway 47500, Malaysia; 2 Department of Biology, Chemistry and Environmental Sciences, College of Arts and Sciences, American University of Sharjah, University City, Sharjah 269999, UAE; 3 National Institute for Biotechnology and Genetic Engineering, Jhang Rd, 44000 Faisalabad, Punjab, Pakistan

**Keywords:** water monitor lizard, antibiotic resistance, conditioned media, antibacterials, cytotoxicity, LCMS

## Abstract

For the past few decades, there has been limited progress in the development of novel antibacterials. Previously, we postulated that the gut microbiota of animals residing in polluted environments are a forthcoming supply of antibacterials. Among various species, the water monitor lizard is an interesting species that feeds on organic waste and the carcass of wild animals. Gut microbiota of the water monitor lizard were sequestered, identified and cultivated in RPMI-1640 to produce conditioned medium (CM). Next, the antimicrobial properties of CM were evaluated versus a selection of Gram-negative (*Escherichia coli* K1, *Serratia marcescens, Pseudomonas aeruginosa, Salmonella enterica* and *Klebsiella pneumoniae*) and Gram-positive bacteria (*Streptococcus pyogenes,* methicillin-resistant *Staphylococcus aureus*, and *Bacillus cereus*). CM were partially characterized by heat inactivation at 95 °C for 10 min and tested against *P. aeruginosa* and *S. pyogenes*. CM were also tested against immortalized human keratinocytes (HaCaT) cells lines. The results demonstrated that gut microbiota isolated from water monitor lizard produced molecules with remarkable bactericidal activities. To determine the identity of the active molecules, CM were subjected to Liquid Chromatography-Mass Spectrometry. Several molecules were identified belonging to the classes of flavonoids, terpenoids, alkaloids, polyhydroxy alkaloids, polyacetylenes, bisphenols, amides, oxylipin and pyrazine derivatives with known broad-spectrum antimicrobial, anti-tumour, anti-oxidant, anti-inflammatory, and analgesic attributes. Furthermore, the detailed analysis of these molecules could lead us to develop effective therapeutic antibacterials.

## 1. Introduction

Infectious diseases have remained as a prominent reason of mortality worldwide since ancient times [1,2]. Nonetheless there has been substantial progress in the field of antimicrobial innovation, chemotherapy and healthcare [3]. Infectious diseases are significant particularly in the developing world, where they contribute to approximately 45% of all fatalities [4]. Furthermore, emergence of drug resistance is exacerbating the situation, contributing to 23,000 deaths in the USA [5,6] and 25,000 deaths in Europe alone [7]. Henceforth, ascertaining novel molecules that target bacterial infections is imperative. Secondary metabolites isolated from numerous microbes have been proven effective against pathogenic bacterial infections [8]. The increasing incidence of multiple-drug-resistant (MDR) strains such as those of the ESKAPE (*Enterococcus faecium, Staphylococcus aureus, Klebsiella pneumoniae, Acinetobacter baumannii, Pseudomonas aeruginosa*, and *Enterobacter* spp.) is a cause for apprehension [8,9]. The assessment of novel bioactive antimicrobials coming from untapped, overlooked and unique natural supplies will produce useful leads to develop novel drugs [3].

Our previous research has shown that the gut bacteria of animals/pests living in polluted environments produces effective antibacterial molecule(s) [10,11]. Among various species, *Varanus salvator* (water monitor lizard) represents an important species living in unhygienic conditions throughout Southeast Asia [12]. These species have been utilised for medicinal purposes in their local context, and have been observed to feed on garbage, human-discarded food and the carcasses of wild animals such as the pig [12,13]. The rationale of the present study was to ascertain if the gut bacteria of the water monitor lizard (WML) has potential to produce antibacterial molecule(s).

## 2. Results

### 2.1. A Plethora of Bacteria Were Isolated from the Gut of Water Monitor Lizard

Several bacteria were isolated from the gut of water monitor lizard and sub-cultured on to nutrient agar plates to acquire pure cultures (Table 1). Bacterial identification was done using microbiological techniques, Analytical Profile Index (API) strips and biochemical assessment, and the results revealed *P. mirabilis*, *A. hydrophila*, *C. freundii*, *E. coli*, *Staphylococcus* sp. and *S. aureus*. Bacteria were cultivated in RPMI-1640 (minimal medium) for 24 h at 37 °C and CM were produced. CM from various bacteria comprised of CM1 (*P. mirabilis*), CM2 (*A. hydrophila*), CM3 (*C. freundii*), CM4 (*E. coli*), CM5 (*Staphylococcus* sp.), CM6 (*S. aureus*), and CM7 (*E. coli* K-12). CM were subsequently assessed against Gram-negative and Gram-positive pathogenic bacteria for bactericidal properties.

### 2.2. Bacterial Sensitivity Profile

Bacteria isolated from the gut of water monitor lizard were subjected to antibiotic sensitivity tests. Among them, *P. mirabilis* was sensitive to ampicillin, augmentin, cefuroxime, ciprofloxacin, ceftriaxone, gentamicin and imipenem. *A. hydrophila* exhibited resistance to imipenem, cefoperazone and tazocin although sensitive to amikacin, ceftazidime, ciprofloxacin, gentamicin, tobramycin and netilmicin. *C. freundii* displayed resistance to ampicillin, augmentin, cefuroxime and ceftriaxone but was sensitive to amikacin, ciprofloxacin, gentamicin, imipenem tobramycin and netilmicin. *E. coli* did not show any resistance but was found sensitive to ampicillin, augmentin, ceftriaxone, ciprofloxacin, cefuroxime, gentamicin and imipenem. *Staphylococcus* spp. was resistant to fusidic acid, methicillin and penicillin but sensitive to erythromycin, rifampicin, tetracycline and linezolid. On the other hand, *S. aureus* was sensitive to erythromycin, methicillin, fusidic acid, penicillin, tetracycline, rifampicin, teicoplanin, azithromycin and linezolid respectively.

### 2.3. Gut Bacteria of Water Monitor Lizard Exhibited Bactericidal Properties Against Pathogenic Gram-Positive and Gram-Negative Bacteria

**Figure 1:**
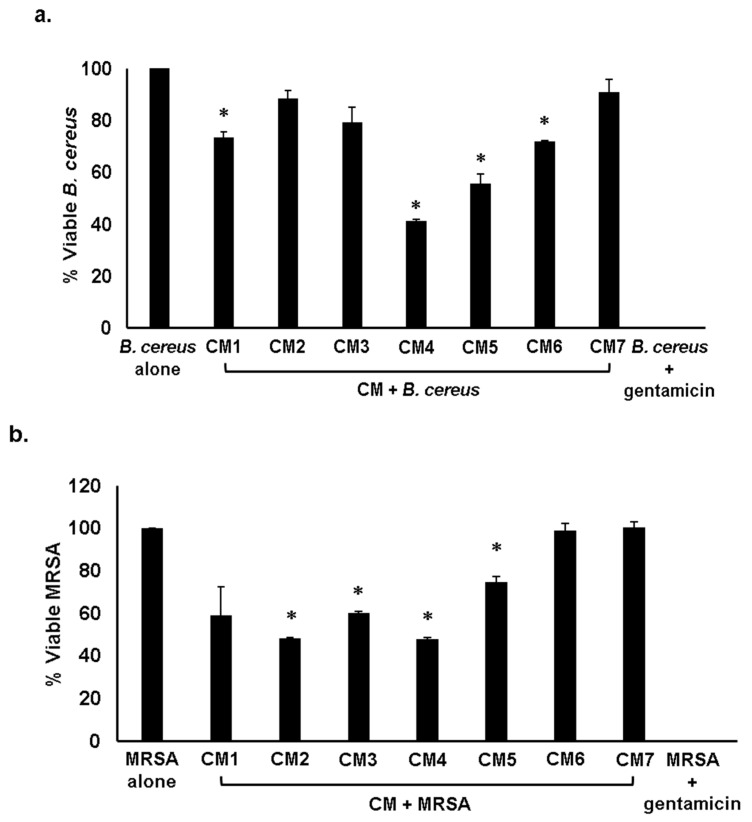

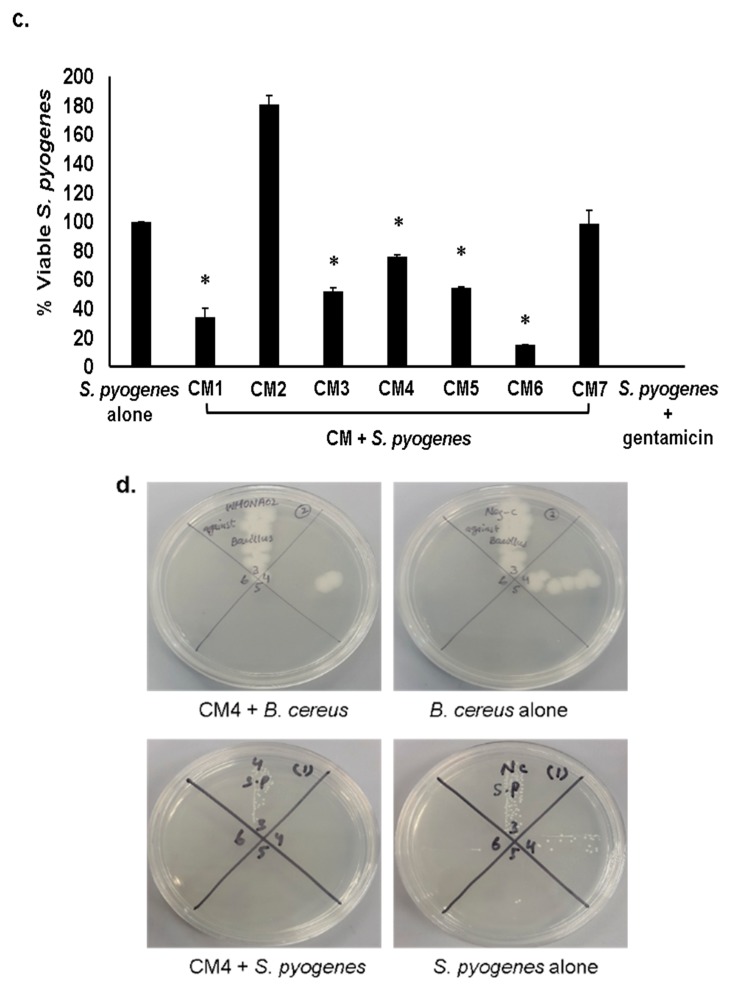
Conditioned medium (CM) from water monitor lizard (WML) gut bacteria exhibited significant bactericidal activities against selected Gram-positive pathogenic bacteria. Briefly, 1 × 10^6^ bacteria were mixed with CM from WML gut bacteria and culture was incubated at 37 °C for 2 h. Next, the culture was serially diluted and plated on nutrient agar plates. Plates were incubated at 37 °C for 24 h and bacterial colonies were enumerated. The data is expressed as the mean ±standard error of several independent experiments performed in duplicate. *P* values were determined using student’s *T*-test, two-tailed distribution, (*) is *P* ≤ 0.05. Bacteria incubated with phosphate-buffered saline (PBS) and gentamicin (100 µg/mL) were taken as negative and positive controls. (**a**) CM tested against *B. cereus*, (**b**) against MRSA (**c**) against *S. pyogenes* and (**d**) representative effects of CM against *B. cereus* and *S. pyogenes*. CM1 is *Proteus mirabilis*, CM2 is *Aeromonas hydrophila*, CM3 is *Citrobacter freundii*, CM4 is *E. coli*, CM5 is *Staphylococcus* sp., CM6 is *Staphylococcus aureus* and CM7 is *E. coli* K-12.

**Figure 2:**
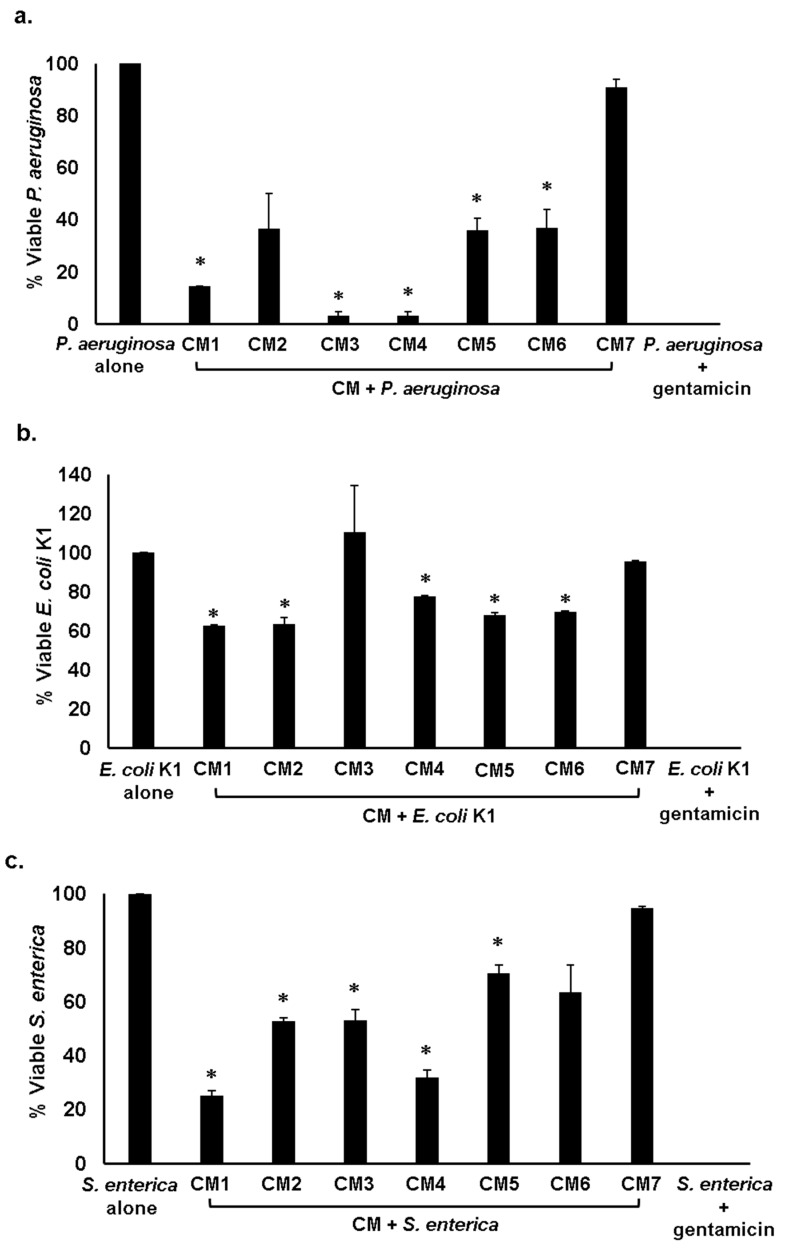

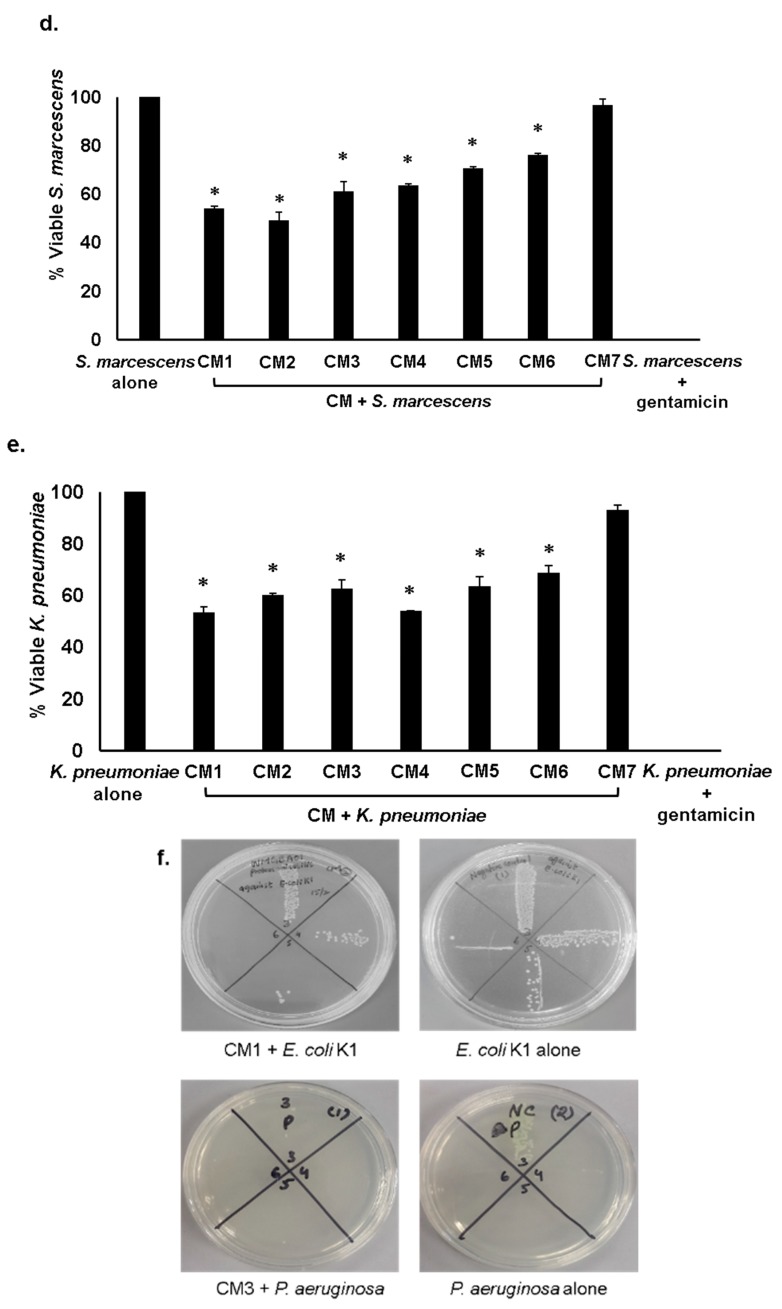
Conditioned medium (CM) from water monitor lizard (WML) gut bacteria exhibited significant bactericidal activities against selected Gram-negative pathogenic bacteria. Briefly, 1 × 10^6^ bacteria were mixed with CM from WML gut bacteria and culture was incubated at 37 °C for 2 h. Next, the culture was serially diluted and plated on nutrient agar plates. Plates were incubated at 37 °C for 24 h and bacterial colonies were enumerated. The data is expressed as the mean ± standard error of several independent experiments performed in duplicate. *P* Values were determined using student’s T-test, two-tailed distribution, (*) is *P* ≤ 0.05. Bacteria incubated with PBS and gentamicin (100 µg/mL) were taken as negative and positive controls. (**a**) CM tested against *E. coli* K1, (**b**) against *P. aeruginosa* (**c**) against *S. enterica* (**d**) against *S. marcescens*, (**e**) against *K. pneumoniae* and (**f**) representative effects of CM against *E. coli* K1 and *P. aeruginosa*. CM1 is *P. mirabilis*, CM2 is *A. hydrophila*, CM3 is *C. freundii,* CM4 is *E. coli*, CM5 is *Staphylococcus* sp., CM6 is *S. aureus* and CM7 is *E. coli* K-12.

In the microbial world, microorganisms produce substances to compete with other organisms for nourishment and space. The strains that produce these secretions are immune to these molecules [4,10]. Entire CM except CM2, CM3 and CM7 displayed significant antibacterial activities against *B. cereus*, (*p* < 0.05 using student’s *t*-test, two-tailed distribution) (Figure 1a and Table 2). When tested against methicillin resistant *Staphylococcus aureus* (MRSA), every CM except CM1, CM6 and CM7 showed substantial bactericidal effects (*p* < 0.05) (Figure 1b and Table 2). For *S. pyogenes*, all CM apart from CM1, CM2 and CM7 displayed potent antibacterial properties (Figure 1c and Table 2) (*p* < 0.05). When the CM were evaluated against Gram-negative bacteria, all CM excluding CM2 and CM7 demonstrated substantial bactericidal properties against *P. aeruginosa* (*p* < 0.05) (Figure 2a and Table 2). Moreover, all CM excluding CM3 and CM7 displayed remarkable antibacterial effects against *E. coli* K1, (*p* < 0.05) (Figure 2b and Table 2). When the CM were tested against *S. enterica*, the results disclosed that all CM except CM6 and CM7 portrayed noteworthy antibacterial properties (Figure 2c and Table 2) (*p* < 0.05). When investigated against *K. pneumoniae* and *S. marcescens*, each CM with the exception of CM7 presented significant bactericidal properties (*p* < 0.05) (Figure 2d,e and Table 2).

### 2.4. Antibacterial Properties of CM Were Heat-Resistant

**Figure 3:**
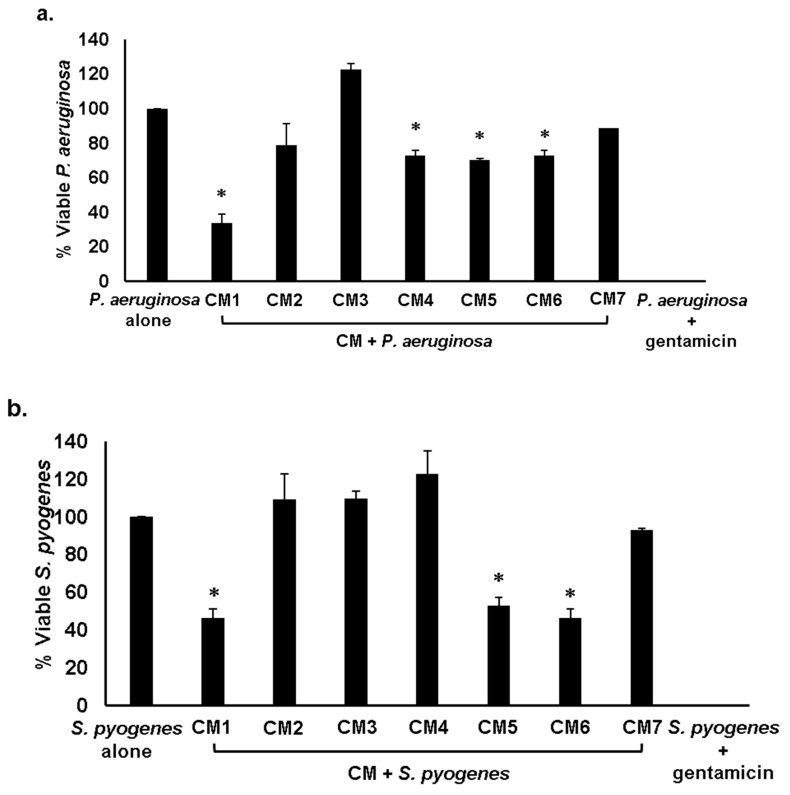

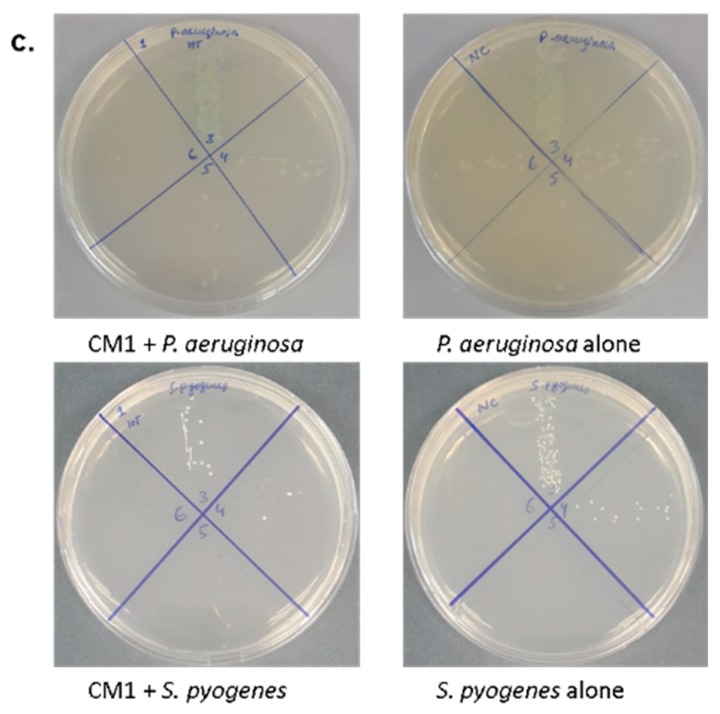
Heat treated CM from water monitor lizard (WML) gut possessed antibacterial properties against *S. pyogenes* and *P. aeruginosa*. Briefly, 1 × 10^6^ bacteria were incubated with heat-inactivated CM at 37 °C for 2 h. Next, the culture was serially diluted, plated on nutrient agar and plates were incubated at 37 °C for 24 h. Next day, bacterial viability was measured by enumerating viable bacterial colonies. All the experiments were performed several times in duplicate and *P* values were determined using student’s t-test. (*) represents *P* ≤ 0.05. (**a**) CM tested against *P. aeruginosa* (**b**) against *S. pyogenes*, and (**c**) representative effects of CM against *P. aeruginosa* and *S. pyogenes*.

Conditioned medium prepared from water monitor gut bacteria were exposed to high temperature of 95 °C for 10 min and bactericidal effects were assessed against *P. aeruginosa* and *S. pyogenes*. The results disclosed that when CM antibacterial activity was established against *P. aeruginosa*, all CM excluding CM2, CM3 and CM7 showed antibacterial properties (Figure 3a) whereas in case of *S. pyogenes* CM1, CM5 and CM6 exhibited potent bactericidal effects (Figure 3b) suggesting that active molecules are heat-resistant. 

### 2.5. Conditioned Medium Showed Limited Cytotoxic Effects on Human Cells

**Figure 4:**
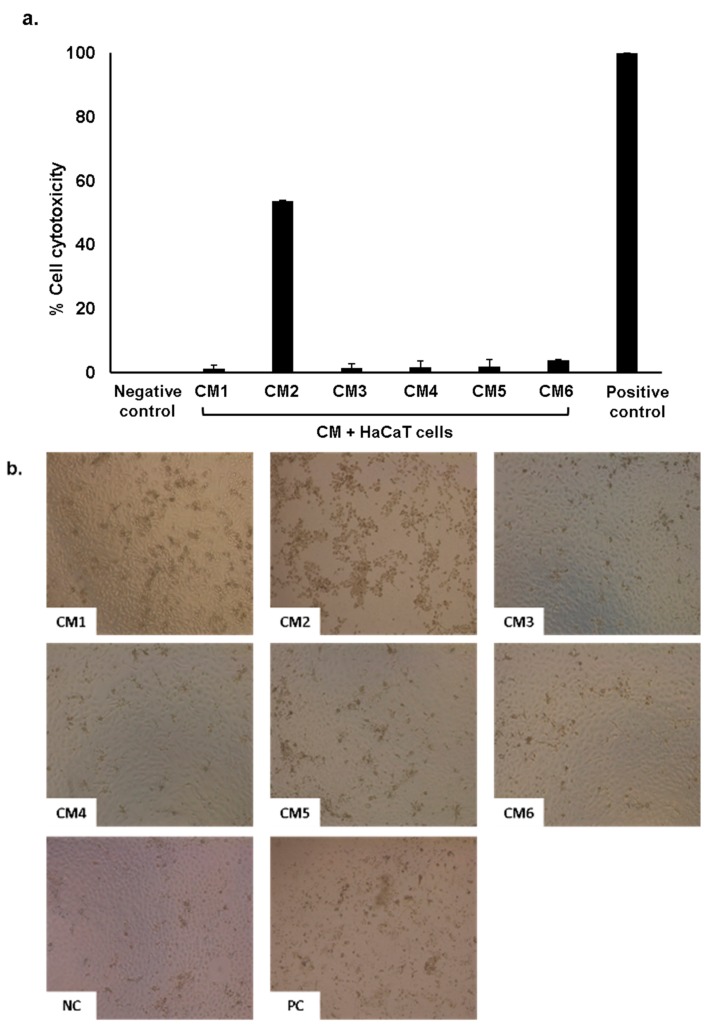
Host cell cytotoxicity assays of conditioned medium against HaCaT cell lines. Briefly, HaCaT cells were grown to (80–90%) confluency and then exposed to 100 µL of CM in a 96 well plate for 24 h at 37 °C in the presence of 5% CO_2_ and 95% humidity. Next day, Lactate dehydrogenase LDH released was determined as described in material and methods. (**a**) All CM tested were non-toxic, except CM2 that exhibited toxicity against HaCaT cells. (**b**) Representative images of cell monolayer incubated with CM.

Conditioned medium prepared from gut bacteria were tested to determine cell cytotoxicity against HaCaT cell lines. HaCaT cells were grown in 96 well plates and assays were performed as discussed in methods. The results showed that among all CM tested, only CM2 exhibited 53% cytotoxicity as compared to positive control i.e., 100%. All other CM did not show cytotoxic effects (Figure 4a,b). As expected, CM7 produced by *E. coli* K-12 showed no effects on human cell cytotoxicity.

### 2.6. Liquid Chromatography-Mass Spectrometry Revealed a Plethora of Compound(s) from CM of Water Monitor Lizard Gut Microbiota 

The results revealed that water monitor lizard gut bacteria produced extensive metabolites/compounds including flavonoids, alkaloids, terpenoids, hydroxylated as well as oxygenated fatty acids and pyrazine derivatives as shown in Supplementary Table S1. These compounds exhibited significant broad-spectrum antibacterial properties against a panel of Gram-positive and Gram-negative bacteria. In total, 601 molecules were determined, out of these, 73 were identified in CM from water monitor lizard gut bacteria when the data were processed using Metlin_AM_PCDL-N-170502.cdb search database. Furthermore, out of these 73, a few of the molecules were similar produced by different bacteria of WML gut therefore, similar compounds have been eliminated and the number of molecules remained were 54 as shown in (Supplementary Table S1). These compounds were separated based on their mass to charge (*m/z)*) ratio. The identified compounds were examined in Scifinder database to elucidate reported biological activity. Among the identified compound(s) several demonstrated biological activities. For example, compounds 1, 4, 5, 8, 12, 13, 22, 27, 33, 34, 38, 39, 49, 51 and 53 were shown to have antibacterial properties against Gram-positive and Gram-negative bacteria including *Mycobacterium smegmatis*, *M. avium*, *Micrococcus luteus*, *Enterococcus faecalis*, *S. aureus* and *S. epidermidis*, *E. faecium*, *B. subtilis*, *P. aeruginosa*, *E. coli*, *P. reinekei* and *H. pylori*. Compounds 3, 16 and 39 were shown to possess anti-inflammatory activity [14,15,16,17,18,19,20,21,22]. Compounds 11, 13, 36, 39, 48 and 54 possess antifungal activity where compounds 15 and 16 possess anti-oxidant activities [23,24,25,26,27]. Moreover, there was no biological activity reported for compounds 2, 3, 7, 9, 26, 28, 29, 40, 42 and 47.

## 3. Discussion

Over the past few decades, pathogenic bacteria acquired matchless resistance to clinically available drugs [28]. This constant evolution in bacteria drives an unceasing demand for novel antimicrobial agents [9]. Microbes compete with each other by producing bioactive molecules in their surroundings. These microbial derivatives have been of immense worth in contemporary medicine [10]. For example, a novel antibiotic Merochlorin A was sequestered from a marine-derived actinomycetes strain CNH189 that exhibited remarkable bactericidal properties against multi drug-resistant Gram-positive bacteria, comprising MRSA and *C. dificile* [9]. Similarly, alkaloids, flavonoids, polyketones, quinols, peptides, terpenoids and steroids have been isolated from the endophytic bacteria i.e., *Streptomyces* sp. which are effective against MDR bacteria. These molecules showed antibacterial activities with no toxicity to human cells [29,30]. Lassomycin, a ribosomally encoded cyclic peptide with a unique structure was isolated from an uncultured bacterium i.e., *Lentzea kentuckyensis* species. This peptide has exceptional antibacterial activities against a range of *M. tuberculosis* strains, comprising multiple-drug-resistant and extremely drug-resistant isolates [31]. Similarly, *Actinobacteria* was isolated from farms soil in Egypt. The crude extracts of this bacteria exhibited antibacterial activities against *B. cereus*, *S. aureus*, *E. coli*, *K. pneumoniae*, *P. aeruginosa*, *S. typhi* bacteria and antifungal activities against *A. flavus*, *A. niger* and *C. albicans*. Atta [32], isolated a nucleotide antibiotic Tunicamycin from *Streptomyces torulosus*. This antibiotic was active against a range of Gram-positive (*B. pumilus, B. subtilis, M. lutea, and S. aureus*) and Gram-negative (*E. coli, K. pneumonia*, and *P. aeruginosa*) bacteria, yeast (*Saccharomyces cerevisiae*) and other fungi like *Alternaria alternata*; *A. flavus, A. fumigatus, A. niger, B. fabae, C. albicans, F. oxysporum; P. chrysogenium* and *R. solani* [32]. 

In the present study we isolated gut bacteria from water monitor lizard, identified and cultured it in a minimal media (RPMI) to prepare CM. The CM were examined against several Gram-positive and Gram-negative bacteria to determine their antibacterial activities. Finally, the CM were tested for their cytotoxicity against HaCaT cell lines. The CM from *A. hydrophila* (CM2) exhibited cytotoxicity against HaCaT cells. This is likely because this bacterium secretes cytotoxic enterotoxins that binds to human, yeast proteins and human intestinal epithelial cell line (HT-29) and induced apoptosis [33]. These findings are supported by Chopra et al. [34] and Krzymińska et al. [35], which showed *Aeromonas* species produce enterotoxins, cytotonic and cytotoxic toxins that cause HEp-2 cell death, lyse red blood cells and abolish tissue culture cell lines. The CM exhibited robust bactericidal activities against selected pathogenic bacteria. Most of the CM showed antibacterial effects even after heat inactivation of these CM at 95 °C for 10 min. This further suggests that the active molecules could be possibly small secondary metabolites. The results from LCMS showed several secondary metabolites with antibacterial properties including flavonoids, alkaloids, terpenes, oxygenated fatty acids, hydroxylated fatty acids and pyrazine derivatives. Similar compounds have been reported for example, 2,5-Dihydroxymethyl-3, 4-dihydroxypyr- rolidine (DMDP) exhibited antiparasitic activity against plant parasite nematode [36]. Here for the first time, we identified the same compound from WML gut bacteria which suggests that bacteria from gut have the capability to produce anti-nematodal molecules. Similarly, Dehydrocurdione is found in turmeric [37], while the same compound has been isolated from gut bacteria with significant antibacterial activity against *B. subtilis* in the current study. Littlefield-Wyer et al. [38] identified 10-Hydroxymyristic acid from aquatic microorganisms, while similar molecule is shown to be synthesized by gut bacteria in this study. Several identified molecules with no reported biological activities are S-Methyl-1-thio-D-glycerate, omega-Hydroxymoracin N, 1,3,8-Trihydroxy-4-methyl-2,7-diprenylxanthone, Desmethyl-maprotiline glucuronide, Desmethyl-nortriptyline glucuronide, (3b,21b)-12-Oleanene-3,21,28-triol 28-[arabinosyl-(1->3)-arabinosyl-(1->3)-arabinoside], Chondrilla-sterol 3-[glucosyl-(1->2)-glucosyl-(1->2)-glucoside], 4-Methyl-dibenzothiophene, 1,2-Epoxy-3,4-butanediol 4-methane-sulfonate and 1R,2R)-3-oxo-2-pentyl-cyclopentanehexanoic acid. Further identification, characterization and their functional studies could be a major breakthrough in development of novel drugs against multi-drug-resistant bacteria. In future studies, individual compound(s) will be isolated and screened against MDR bacteria and human cell lines to determine their translational value in therapy.

## 4. Materials and Methods

### 4.1. Bacterial Cultures

Table 3 indicates the bacteria that were utilised in the present study. These include the neuropathogen *E. coli* K1, *S. pyogenes*, *P. aeruginosa*, *B. cereus*, and *K. pneumoniae*. Methicillin-resistant *Staphylococcus aureus* (MRSA), and the non-pathogenic *E. coli* K-12 (Table 3). MRSA was segregated from blood cultures of sepsis patients, attained from the Luton and Dunstable NHS Foundation Trust, Luton, England, UK. Neuropathogenic *E. coli* K1 was originally accessed from the cerebrospinal fluid (CSF) of a meningitis patient. Other bacteria used in this study are derived from clinical samples and available upon request including *S. pyogenes*, *P. aeruginosa*, *K. pneumoniae*, and *B. cereus*. All bacterial isolates were cultivated aerobically at 37 °C in nutrient broth overnight preceding experiments.

### 4.2. Dissection of Water Monitor Lizard 

The use of animals was permitted by the Sunway University Research Ethics Committee, SUREC 2017/042. We also confirm that all experiments were performed in accordance with relevant guidelines and regulations as previously described [11]. All dissecting instruments were sterilized prior to experiments. All surgical instruments were sanitized at the surface with 70% alcohol throughout the dissection. Water monitor lizard was dissected along the middle of the abdominal cavity and the entire gut was then separated aseptically. Bacteria were sequestered from the gut using cotton swabs and overlaid on blood agar plates. Next, plates were kept for 24 h at 37 °C. Distinct bacterial colonies were enumerated based on colour, appearance, texture and shape onto blood agar plates. Dissimilar colonies were grown on nutrient agar plates and kept overnight at 37 °C. Next, recognition of bacterial colonies was done using Analytical profile index (API) along with biochemical test including oxidase, coagulase and catalase [39].

### 4.3. Preparation of Bacterial Conditioned Medium

RPMI-1640 medium (minimal medium) was used for the preparation of conditioned media (CM). Single bacterial colonies were grown in 50 mL of RPMI and cultures were kept for 24 h at 37 °C. Next, cultures were subjected to centrifugation at 10,000× *g* for 50 min at 4 °C. Supernatants were accumulated and filter-sterilized through a 0.22 µm pore size filter. CM were deposited at −80 °C pending use.

### 4.4. Antibacterial Assays 

Antibacterial assays were accomplished as formerly depicted [10,11]. Briefly, for cultures grown overnight, the optical density was diluted to set at 0.22 at 595 nm [this is equivalent to roughly 1 × 10^8^ colony forming unit (c.f.u.) per mL]. Next, 1 × 10^6^ bacterial c.f.u (10 µL) were treated with 100 µL of CM and the ultimate volume was attuned to 200 µL with phosphate-buffered saline (PBS) and kept at 37 °C for 2 h. Next, serial dilution of cultures in distilled water was employed, with subsequent plating on nutrient agar plates. Plates were kept overnight at 37 °C. Any bacterial colonies were quantified the next day. PBS alone and *E. coli* K-12 CM were prepared as negative controls, whereas gentamicin (100 µg per mL) was utilised by way of positive control. For some trials, CM was heat-inactivated for 10 min at 95 °C as explained beforehand. Following heat inactivation, CM was utilised for antibacterial assays as detailed above. For partial identification of active molecules, conditioned media were inactivated by heating at 95 °C for 10 min [4,10]. Next, their antibacterial activities were established against *P. aeruginosa* (Gram-negative) and *S. pyogenes* (Gram-positive).

### 4.5. Host Cell Cytotoxicity Analyses 

Host cell cytotoxicity analyses were accomplished as designated earlier [40,41]. Assays were achieved in 96 well plates comprising human keratinocytes (HaCaT) cells monolayers. The cells were treated with 100 µL of CM from the water monitor lizard’s gut bacteria and kept for 24 h in the presence of 5% CO_2_ and 95% humidity at 37 °C. Next day, Triton X-100 (final conc. 0.1%) was utilised as a positive control and plates were kept for 45–60 min at 37 °C. Following this incubation, an equal amount of supernatant (containing Lactate Dehydrogenase enzyme) from each well was mixed with equal amount of LDH kit reagents (Cytotoxicity Detection kit; Roche Diagnostics, Indianapolis, IN, USA) and cytotoxic activity was established by the estimation of LDH released from HaCaT cells: cytotoxicity (%) = (sample value–negative control value)/(positive control value–negative control value) × 100. For negative controls, HaCaT monolayers were grown in RPMI only and 100% LDH release was determined by lysing HaCaT cells with 0.1% of triton X-100.

### 4.6. Mass Spectrometric Analysis of Culture Supernatant

Mass spectrometric analysis was performed to identify secondary metabolites of water monitor lizard gut bacteria [42]. Bacteria were inoculated in RPMI-1640 medium (200 mL) and retained for 24 h with continuous shaking, at 37 °C. Bacterial cultures were centrifuged for 60 min at 10,000× *g*. The CM were extracted in chloroform (1:3) and the organic layer (chloroform) was attained and evaporated under reduced pressure using rotary evaporation. Residues were dissolved in 2 mL LCMS grade methanol and subjected to LCMS/MS analysis with the mass spectrometer (Agilent 1290 Infinity LC system coupled to Agilent 6520 Accurate-Mass Q-TOF mass spectrometer) with dual Electrospray ionization (ESI) source. The chromatographic separation was performed using C_18_ column (Agilent Zorbax Eclipse XDB-C_18_, Narrow-Bore 2.1 × 150 mm, 3.5 micron). The temperature of column was 25 °C as stationary phase and the mobile phase consisted of 0.1% formic acid in water (solvent A) and 0.1% formic acid in Acetonitrile (solvent B). Samples were filter sterilized and injected through direct syringe pump with a flow rate of 0.5 mL min^−1^ preceding analysis. Positive and negative total ion full scan mode (mass scan range *m/z* 50–2000) were utilised to scan the samples with capillary voltage of 4.0 kV. The sheath gas flow (N2) was 30 arbitrary units, and the temperature of the capillary was 300 °C in both scan modes. Collision induced dissociation (CID) energy (30 i.e., percentage of 5 V) was employed and designated analytes were fragmented at both positive and negative ion modes. The mass spectrometer spectra for molecules present in CM were run against the Metlin_AM_PCDL-N-170502.cdb search database for the recognition of homologous compounds using Agilent Mass Hunter software. The molecules were subjected to Scifinder software to establish any described biological activities and novelty of identified compounds.

## 5. Conclusions

In summary, we report broad-spectrum antibacterial activities produced by gut bacteria of water monitor lizard. Furthermore, we identified several molecules that may be of clinical significance. These are significant findings and should lead to the development of novel pharmaceutical leads. 

## Figures and Tables

**Table 1:** Bacterial species isolated from the gut of water monitor lizard.

Bacterial Source	Conditioned Medium
*Proteus mirabilis*	CM1
*Aeromonas hydrophila*	CM2
*Citrobacter ferundii*	CM3
*Escherichia coli*	CM4
*Staphylococcus* sp.	CM5
*Staphylococcus aureus*	CM6

**Table 2:** Representation of CM antibacterial activities against Gram-positive and Gram-negative bacterial pathogens.

Conditioned Media	Antibacterial Activities against Gram-Positive Bacteria			Antibacterial Activities against Gram-Negative Bacteria				
	*Bacillus cereus*	MRSA	*Streptococcus pyogenes*	*Escherichia coli* K1	*Klebsiella pneumoniae*	*Pseudomonas aeruginosa*	*Serratia marcescens*	*Salmonella enterica*
CM1	+	−	+	+	+	+	+	+
CM2	−	+	−	+	+	−	+	+
CM3	−	+	+	−	+	+	+	+
CM4	+	+	+	+	+	+	+	+
CM5	+	+	+	+	+	+	+	+
CM6	+	−	+	+	+	+	+	−
CM7	−	−	−	−	−	−	−	−

**Table 3:** Bacteria used in this study.

Bacteria	Strain
Methicillin-resistant *Staphylococcus aureus*	MTCC 381123 (clinical isolate)
*Escherichia coli* K1	MTCC 710859 (clinical isolate)
*Streptococcus pyogenes*	ATCC 49399 (clinical isolate)
*Bacillus cereus*	MTCC 131621 (clinical isolate)
*Pseudomonas aeruginosa*	ATCC 10145 (clinical isolate)
*Klebsiella pneumoniae*	ATCC 13883 (clinical isolate)
*Escherichia coli* K-12	MTCC 817356 (non-clinical isolate)

## Supplementary Materials

The following are available online at https://www.mdpi.com/2079-6382/8/4/164/s1, Figure S1: MS spectrum., Table S1: Compounds identified from gut bacteria of water monitor lizard.
