# Supplementary material for: Gut Bacteria of Water Monitor Lizard (Varanus salvator) Are a Potential Source of Antibacterial Compound(s)
Source: Antibiotics (Basel). 2019 Sep 24;8(4):164. doi: 10.3390/antibiotics8040164 (PMC6963368; doi:10.3390/antibiotics8040164)
Supplement: Supplementary file 1 [file antibiotics-08-00164-s001.pdf]

Figure S1

1. 3',4',5'-Trimethoxyflavone

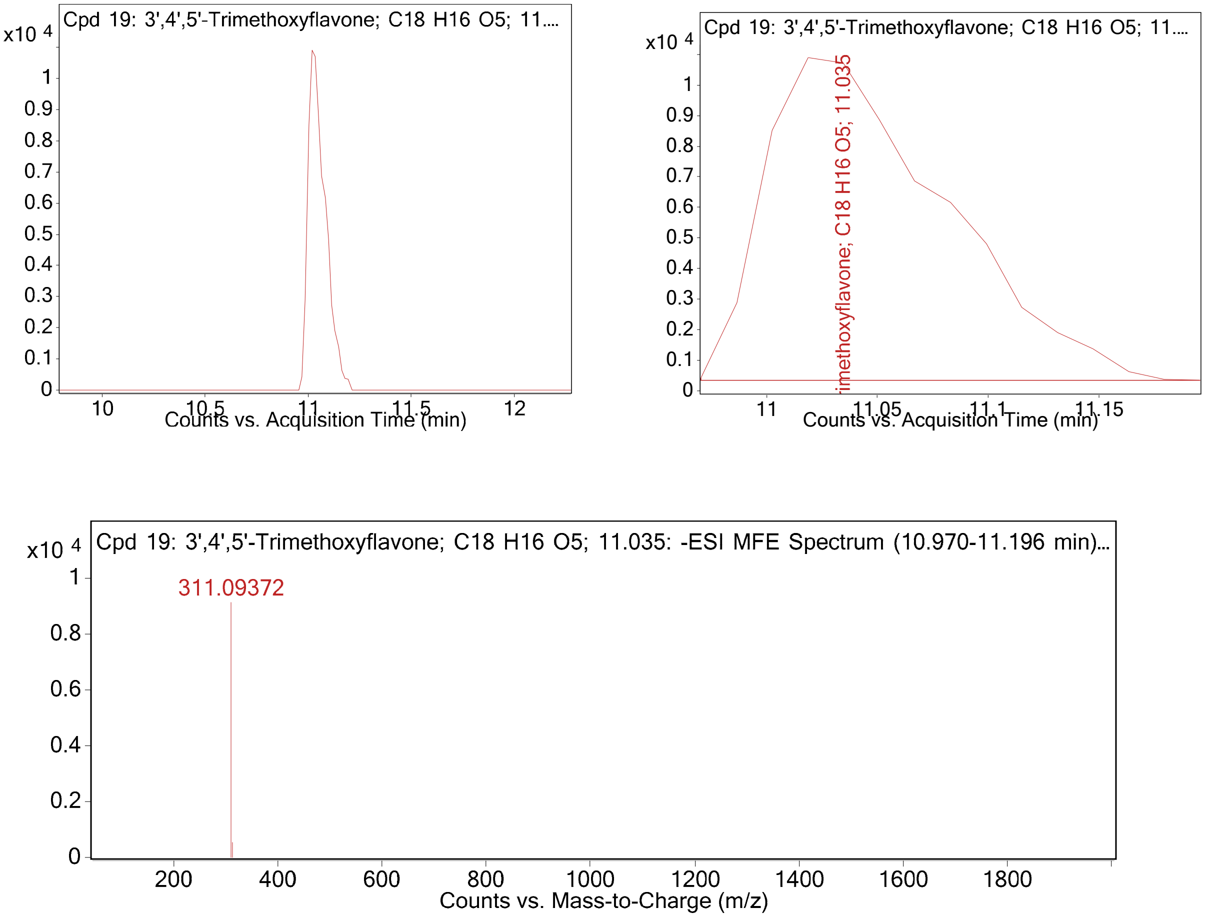

MS spectrum peak list

m/z	z	Abund	Formula	Ion
311.09372	-1	9100.8	$C_{18} H_{15} O_5$	$(M-H)^-$
312.09564	-1	1448	$C_{11} H_{15} N_6 O_3 S$	$(M-H)^-$
313.09493	-1	547.25	$C_{11} H_{15} N_6 O_3 S$	$(M-H)^-$

2. 3'-Geranylchalconaringenin

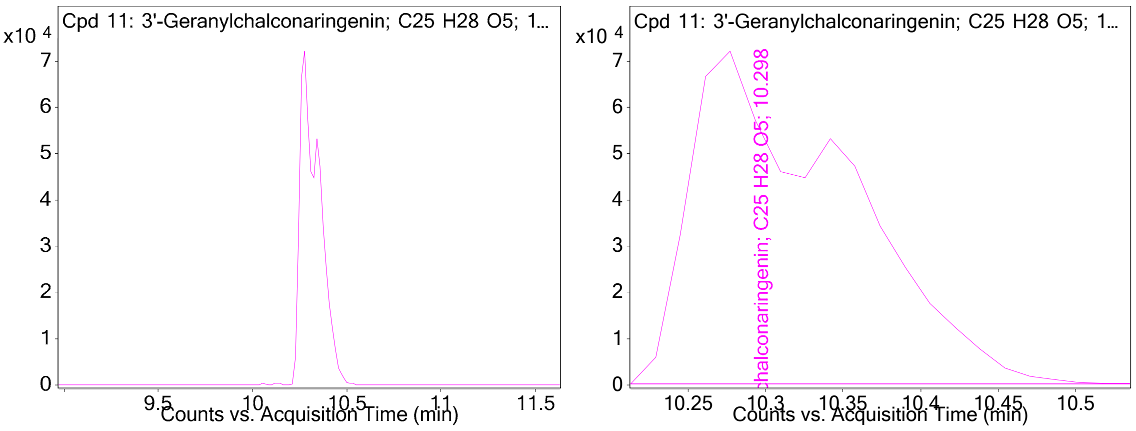

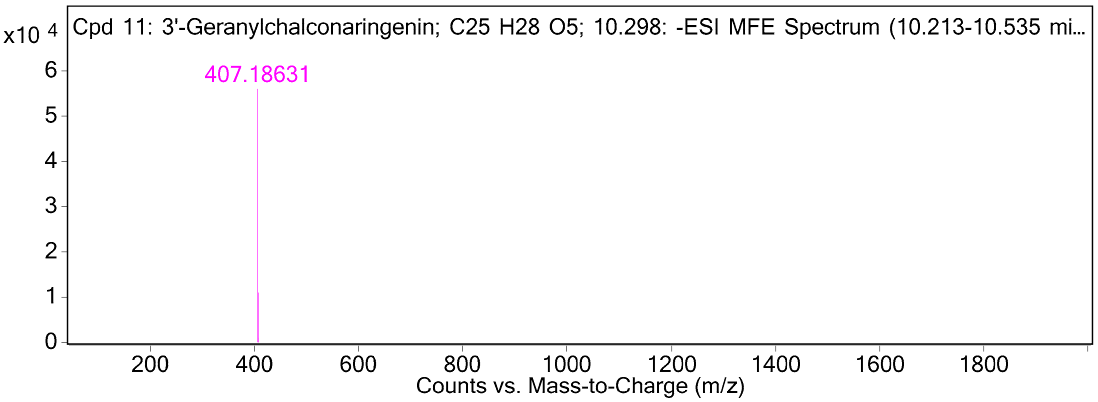

MS spectrum peak list

m/z	z	Abund	Formula	Ion
407.18631	-1	55923.5	$C_{25} H_{27} O_5$	(M-H)-
408.18916	-1	11171.83	$C_{17} H_{31} N_2 O_7 S$	(M-H)-
409.18588	-1	3975.38	$C_{17} H_{31} N_2 O_7 S$	(M-H)-
410.18856	-1	959.04	$C_{17} H_{31} N_2 O_7 S$	(M-H)-

3. Dehydrocurdione

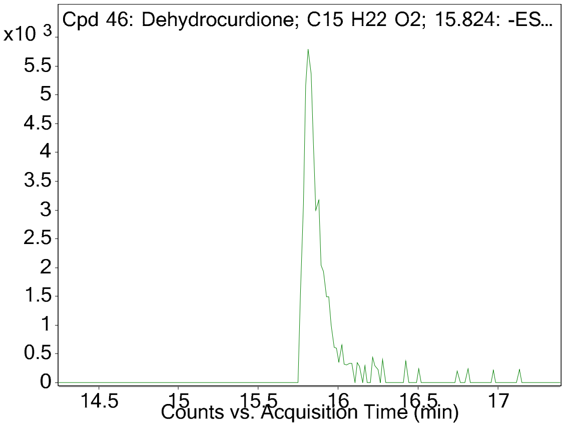

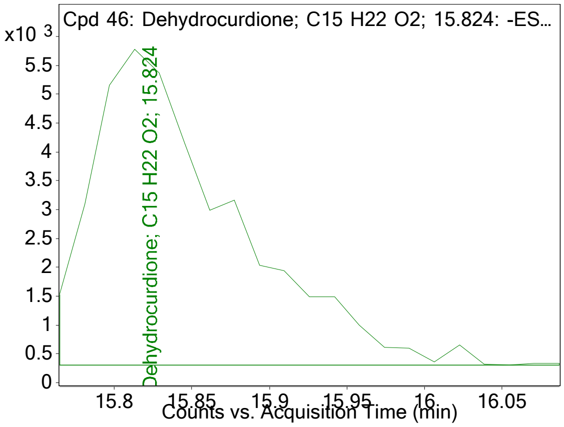

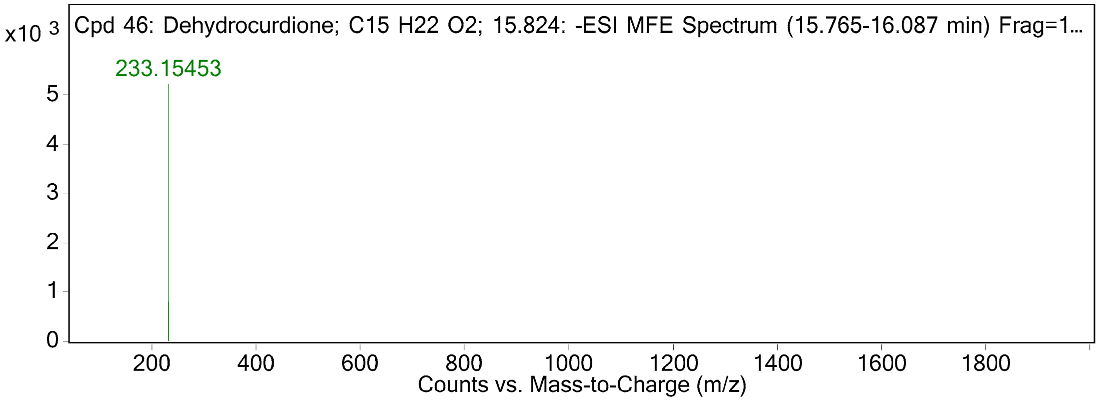

MS spectrum peak list

m/z	z	Abund	Formula	Ion
233.15453	-1	5230.5	C15 H21 O2	(M-H)-
234.15844	-1	807.37	C15 H21 O2	(M-H)-

4. Polidocanol

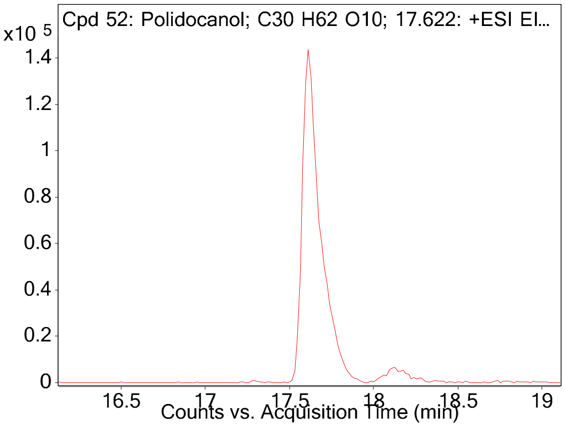

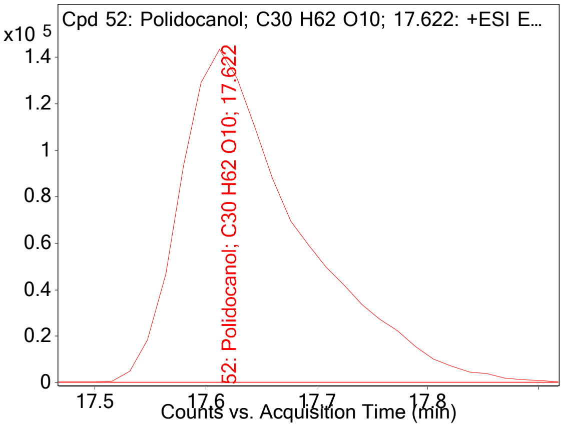

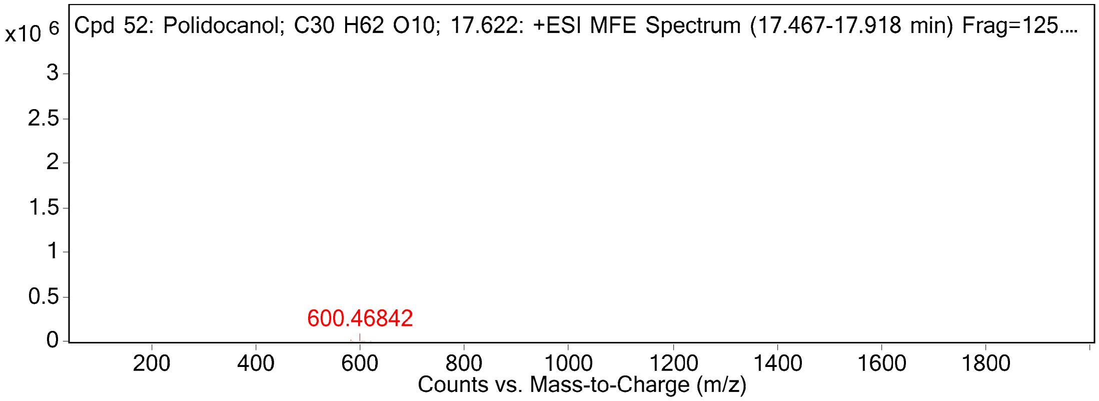

m/z	z	Abund	Formula	Ion
583.4412	1	8820.41	C30 H63 O10	(M+H) +
584.44513	1	3353.26	C30 H63 O10	(M+H) +
585.44541	1	827.22	C30 H63 O10	(M+H) +
600.46842	1	83070.76	C30 H66 N O10	(M+NH4) +
601.4715	1	27828.99	C30 H66 N O10	(M+NH4) +
602.47363	1	6586.78	C30 H66 N O10	(M+NH4) +
603.47744	1	1217	C30 H66 N O10	(M+NH4) +

605.42385	1	7389.64	C30 H62 Na O10	(M+Na) +
606.42801	1	2621.84	C30 H62 Na O10	(M+Na) +
621.39842	1	1056.28	C30 H62 K O10	(M+K) +

Table S1: Compounds identified from gut bacteria of water monitor lizard.

S. No	Compound (s)	Formula	Structure	Reported activities
1.	Lumichrome	$C_{12} H_{10} N_4 O_2$	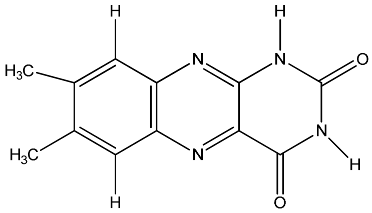	Antimicrobial properties against bacterial pathogens (Ahgilan et al., 2016, Massaro et al., 2014). increased the photosynthetic rates and growth of soybean plants (Khan et al., 2008).
2.	S-Methyl-1-thio-D-glycerate	$C_4 H_8 O_3 S$	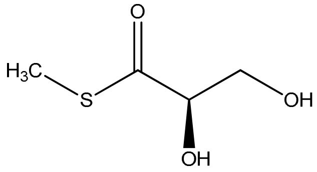	No activity reported.
3.	omega-Hydroxymoracin N	$C_{19} H_{18} O_5$	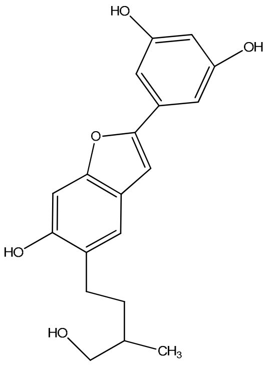	No activity reported. A component in the Anti-inflammatory functional food composition https://patents.google.com/patent/WO2012115469A2/en.

4.	Simulansamide	$C_{22}H_{23}NO_6$	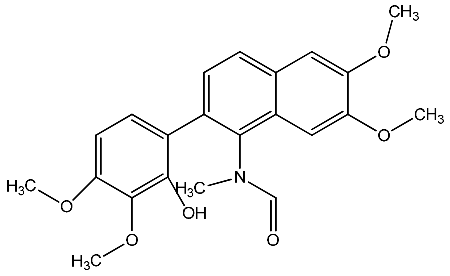	Shows strong inhibition of platelet aggregation (Wu et al., 1996)<br>Exhibit antibacterial activity, cytotoxicity and inhibit DNA isomerase enzyme (Zhou et al., 2011).
5.	Callytriol C	$C_{23}H_{24}O_3$	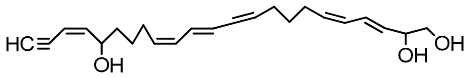	Antimicrobial activities (Tada and Yasuda, 1984).<br>Having antifouling activity against barnacle and metamorphosis promoting activity in ascidians (Pallela and Ehrlich, 2016)
6.	7-Formyldehydrothalicsimidine	$C_{23}H_{25}NO_6$	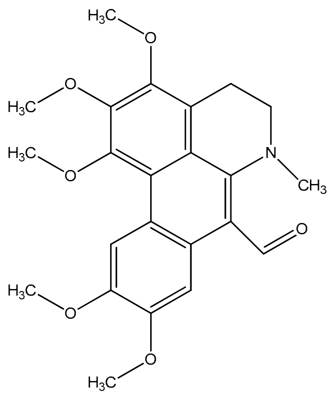	Inhibitor of metalloproteinases (Chang et al., 1998).<br>Exhibit significant inhibition of arachidonic acid, collagen and platelet activating factor-induced platelet aggregation. Inhibition against thrombin-induced platelet

				aggregation (Chang et al., 1998).
7.	1,3,8-Trihydroxy-4-methyl-2,7-diprenylxanthone	$C_{24}H_{26}O_5$	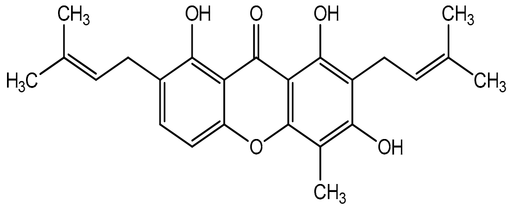	No activity reported. However, 1,3,8-trihydroxy-2,4-dimethoxyxanthone displayed anti-HIV-1 activities (El-Seedi et al., 2010). It is a constituent of the fruit hulls of *Garcinia mangostana* (Yannai, 2003).
8.	3',4',5'-Trimethoxyflavone	$C_{18}H_{16}O_5$	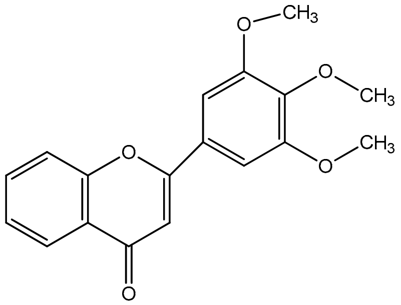	Trimethoxyflavone show antibacterial activity against Gram positive and Gram negative bacteria (FERNANDES et al., 2013).<br>Flavonoids exhibit antibacterial and antioxidant activities (Süzgeç-Selçuk and Birteksöz, 2011).
9.	Desmethylmaprotiline glucuronide	$C_{25}H_{29}NO_6$	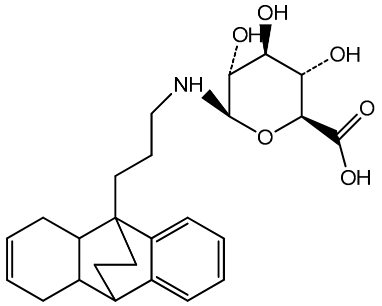	No reported activity. However, Desmethyl Maprotiline itself is antidepressant drug (Rotzinger et al., 1999,

				López-Muñoz and Alamo, 2013).
10.	4,4'-Sulfonyldiphenol	$C_{12} H_{10} O_4 S$	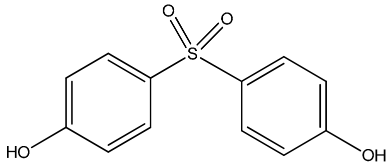	Significant increase TUNEL positive cells in neonatal testis, disruption of the expression of apoptosis, autophagy, and oxidative stress-related factors (Shi et al., 2018). Affect fetal development and increase risk of adverse health consequences during pregnancy (Speidel et al., 2018).
11.	L-Homotyrosine	$C_{10} H_{13} N O_3$	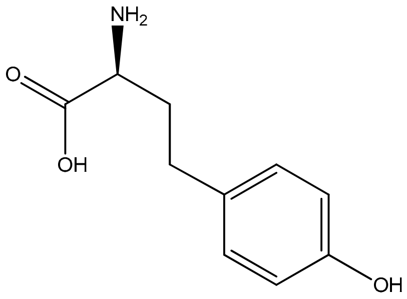	Competitive inhibitors of tyrosine phenol lyase (Do et al., 2016). Derivatives have antifungal activities (Lee et al., 2014, Capobianco et al., 1998).
12.	6-Paradol	$C_{17} H_{26} O_3$	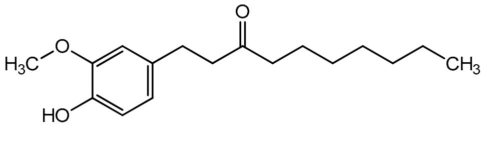	Reduce inflammatory responses in activated BV2 microglia, most effective in neuroinflammation-associated with CNS

				disorders without toxicity (Gaire et al., 2015).<br><br>Antibacterial activities against *Mycobacterium smegmatis* (Galal, 2008) and *Candida albicans* (Abourashed et al., 2007).
13.	trans-2-Hexyl-1-cyclopropaneacetic acid	$C_{11}H_{20}O_2$	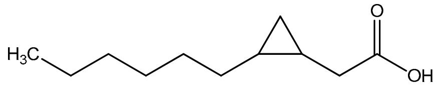	It is a main component of cascarilla essential oil that has antibacterial activity against *Mycobacterium avium* and antifungal activities against some fungi (Opdyke, 1979).<br><br>Acute cytotoxicity in mice (Opdyke, 1979).
14.	Lauryl hydrogen sulfate	$C_{12}H_{26}O_4S$	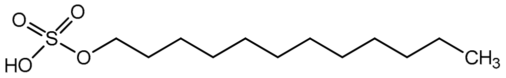	Commonly used as an adjuvant or synergist with antimicrobials and insecticides (Baker and Grant, 2018).<br><br>To control immature mosquitos (Piper and Maxwell, 1971).<br><br>Rapidly biodegradable in the environment (Singer and Tjeerdema, 1993) and

				under laboratory conditions (Leal et al., 1991). Responsible for oxygen deprivation and suffocation (Piper and Maxwell, 1971)
15.	11S-hydroxy-tetradecanoic acid	$C_{14} H_{28} O_3$	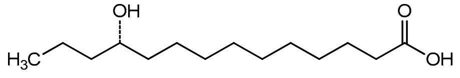	It is an anti-oxidant, cancer preventive and hyper-cholesterolemic (Gomathi Rajashyamala and Elango, 2015).
16.	2-Dodecylbenzenesulfonic acid	$C_{18} H_{30} O_3 S$	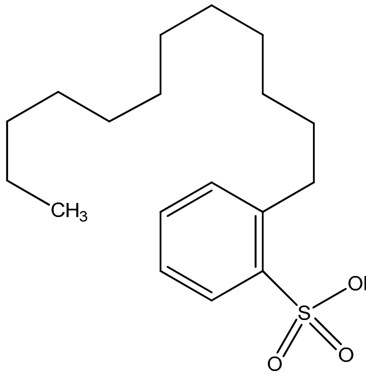	Dodecyl benzene sulfonic acid is extensively applied anionic surfactant (Mimanne et al., 2012). No reported activity itself. A novel, biodegradable, and efficient Brønsted acid catalyst used for the reaction of indoles/4-hydroxy coumarin with aldehydes to obtain a bis(indolyl)methanes/ bis (4- hydroxycoumarin-3- yl)methanes. (Mimanne et al., 2012).

				The coumarin has therapeutic potential for antibiotic, anti-inflammatory, anti-tumor, anti-coagulant, analgesic, anti-HIV, cytotoxic, anti-apoptotic, anti-oxidant and insecticidal activities (Pawar et al., 2013)<br>An emulsifier for agricultural herbicides (Mimanne et al., 2012) .
17.	Tetradecyl sulfate	$C_{14} H_{30} O_4 S$	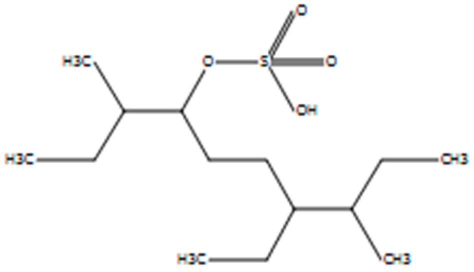	Its sodium salt is used as formulations for treatment of adipose tissue (Dobak, 2017).<br>Treatment of Pyogenic Granuloma (Moon et al., 2005).<br>The infiltrations of sodium tetradecyl sulfate into stromal tissues can cause tissue necrosis (Goldman et al., 1986).

18.	Bis(methylthio) selenide	$C_2 H_6 S_2$ Se	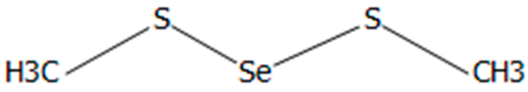	Volatile bis(methylthio)-selenide found in natural elephant garlic and onion oil (Meija and Caruso, 2004). Selenide is the intermediate component in the metabolic pathway of Se in plants (Hudson et al., 2012).
19.	5-Valerolactone	$C_5 H_8 O_2$	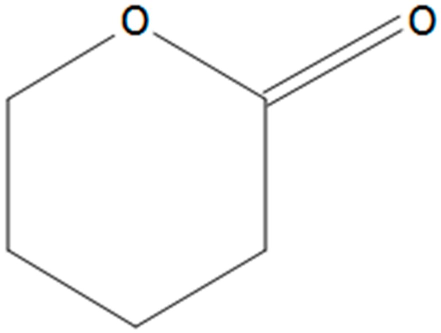	Valerolactone and some analogues exhibit antioxidant activity (Sánchez-Patán et al., 2011).
20.	Cycluron	$C_{11} H_{22} N_2 O$	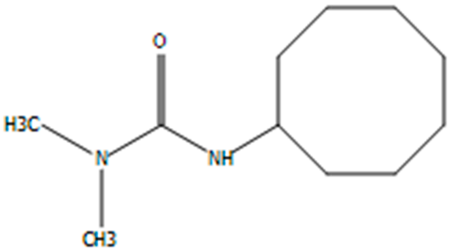	Cycluron has pesticidal activity and effective for the control of monocotyledonous germinating weeds (Matolcsy et al., 1989, Draber and Fujita, 1992).

21.	Atenolol	$C_{14}H_{22}N_2O_3$	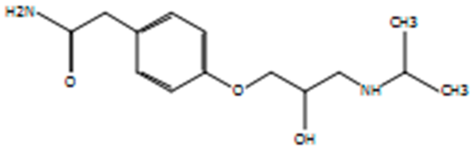	Hydrophilic β-adrenergic blocking agent (AGON et al., 1991).<br>For the treatment of Hypertension and angina (Akarim et al., 2015).<br>Prevent migraines https://www.drugs.com/ monograph /atenolol.html.<br>Antimicrobial activities against *Micrococcus luteus* and *Candida tropicalis* (Gölcü and Yavuz, 2008).
22.	Palmitoleamide	$C_{16}H_{31}NO$	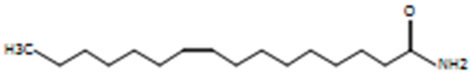	Palmitoleamides are fatty acid amide with antibacterial activities (Zaher et al., 2015)
23.	HClO	Cl H O	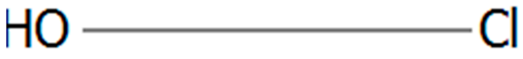	A powerful oxidizer and deproteinizer (Eryilmaz and Palabiyik, 2013).<br>Has good microbicidal activity (Eryilmaz and Palabiyik, 2013).

				Widely used as disinfectant (Kunawarote et al., 2010). Reacts with several biomolecules, especially carbohydrates, amino groups, proteins, thiol, thiol ether, heme as well as overcomes pathogens to fight infections in the body (Kunawarote et al., 2010, Pattison and Davies, 2001, Wang et al., 2007). Important role in bacterial killing (McKenna and Davies, 1988). Play role in host defense by killing pathogens and initiates cell apoptosis (Tian et al., 2016).
24.	N-Acryloylglycine	$C_5H_7NO_3$	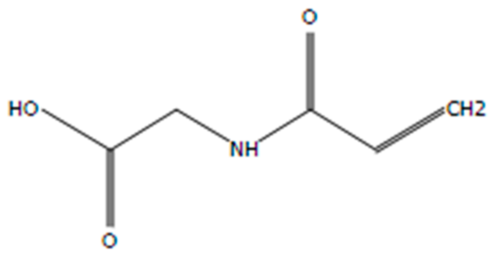	Used in the preparation of hydrogel and as a drug carrier (Deng et al., 2011).

25.	N-Methylcalystegine B2	$C_8 H_{15} N O_4$	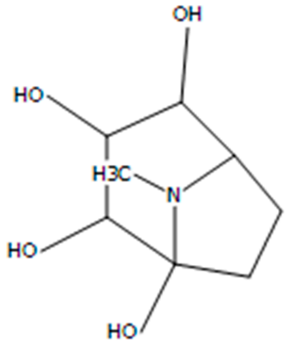	Calystegines are polyhydroxy alkaloids act as α-galactosidase inhibitors (Asano et al., 1997).
26.	Desmethylnortriptyline glucuronide	$C_{24} H_{27} N O_6$	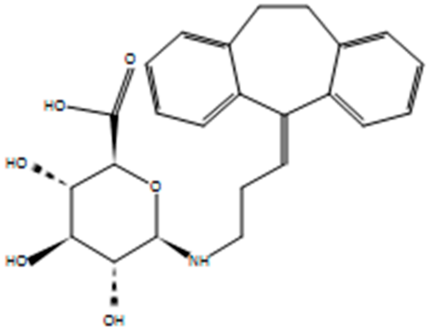	No biological activity reported.
27.	3'-Geranylchalconaringenin	$C_{25} H_{28} O_5$	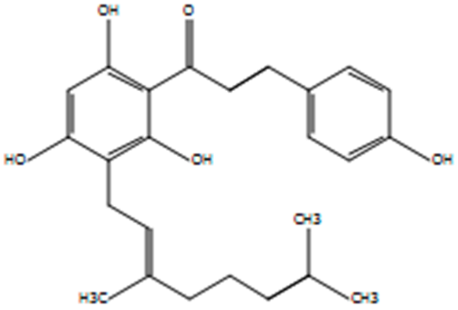	This compound belongs to class of flavonoids (Stevens et al., 1999, Rauha, 2001).<br>The derivatives of this compound exhibit antibacterial activity (Feng et al., 2014).<br>Inhibit α-glucosidase irreversibly and moderately inhibit α-amylase activity (Sun et al., 2017).

28.	(3b,21b)-12-Oleanene-3,21,28-triol 28-[arabinosyl-(1->3)-arabinosyl-(1->3)-arabinoside]	$C_{45}H_{74}O_{15}$	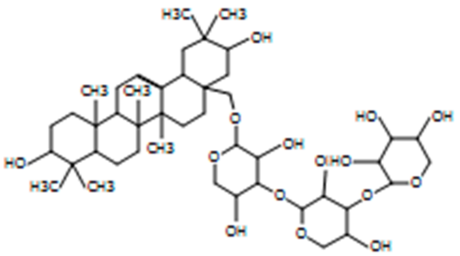	No biological activity reported.
29.	Chondrillasterol 3-[glucosyl-(1->2)-glucosyl-(1->2)-glucoside]	$C_{47}H_{78}O_{16}$	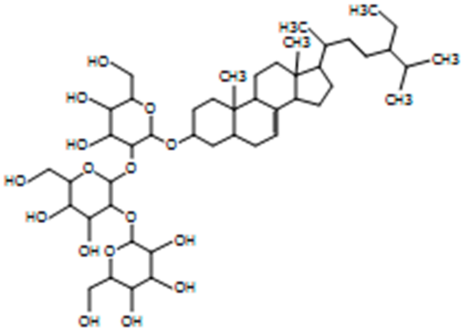	No biological activity reported.
30.	8,8-Diethoxy-2,6-dimethyl-2-octanol	$C_{14}H_{30}O_{3}$	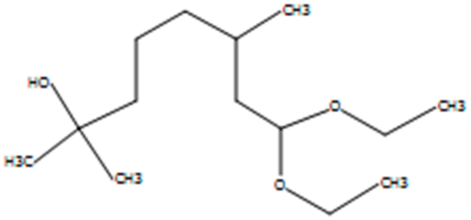	Used in citrus fruit flavourin http://pubchem.ncbi.nlm.nih.gov/compound/hydroxylcitronellal_diethyl_acetal#section=Top. Repellent activities against *Tribolium castanenum* (Weiqing et al., 2009).

31.	Polidocanol	$C_{30}H_{62}O_{10}$	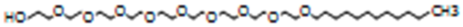	As a sclerosant for varicose veins (Collini, 2000).<br><br>Antibacterial activity against *S. aureus* (Sadick et al., 1996).<br><br>Exhibit excellent performance in improving scalp dryness, itching, micro-inflammation, and in normalizing disturbances of scalp lipids (Schweiger et al., 2013).
32.	Acetylenedicarboxylate	$C_4H_2O_4$	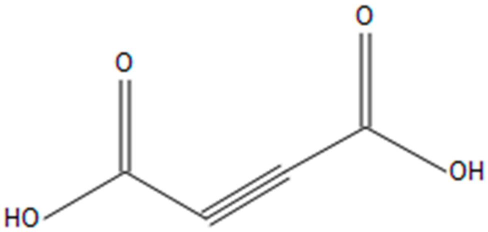	The catabolism of 2-butynedioic acid acts as a precursor of nicotinamide adenine dinucleotide (Heard et al., 1981).

33.	Methimazole	$C_4 H_6 N_2 S$	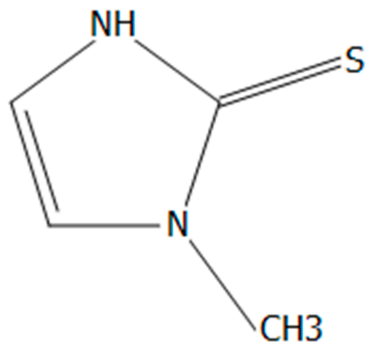	Anti-thyroid drug, normally used to treat Graves' disease, an inhibitor of the enzyme thyroid peroxidase (Sainis et al., 2016, Urquiza et al., 2013). Its derivatives exhibit antibacterial activity against *P. aeruginosa* and *E. coli* (Sainis et al., 2016). Antibacterial activity against *P. aeruginosa, E. coli, E. faecalis, S. aureus* and *S. epidermidis* bacteria (Urquiza et al., 2013).
34.	Pyrazineethanethiol	$C_6 H_8 N_2 S$	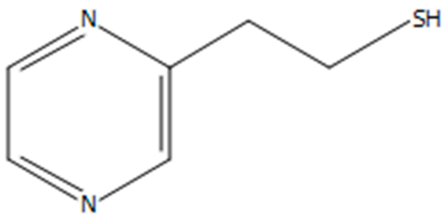	Derivatives of pyrazine inhibiting 50% of the growth of *Streptococcus faecium* and *E. coli* (Bobek and Bloch, 1972).

35.	Salmefamol	$C_{19}H_{25}NO_4$	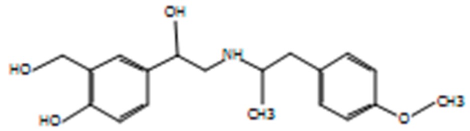	Salmefamol is useful for treatment of respiratory diseases https://www.adooq.com/salmefamol.html. Used as a bronchodilator https://chem.nlm.nih.gov/chemidplus/rn/18910-65-1.
36.	7-hydroxy pelargonic acid	$C_9H_{18}O_3$	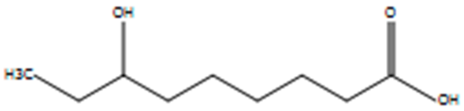	It is a saturated fatty acid used as flavourings, the derivative 4-nonanoylmorpholine is an ingredient in some pepper sprays and has antifungal activity, used as herbicide as well as in the preparation of plasticisers and lacquers https://pubchem.ncbi.nlm.nih.gov/compound/nonanoic_acid#section

37.	Methyl jasmonate	$C_{13}H_{20}O_3$	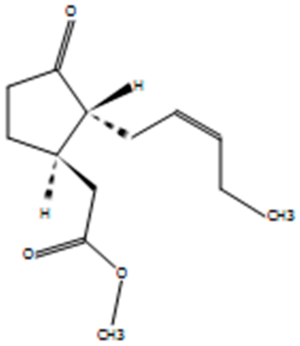	A volatile organic compound used in plant defense and several developmental pathways such as germination of seed, root growth, flowering, fruit ripening, and senescence (Cheong and Do Choi, 2003). Jasmonate are oxylipin, a derivative of oxygenated fatty acid. Exhibit antibacterial activity against *E. coli, P. aeruginosa, S. aureus, S. epidermidis* and *C. albicans* (Andrys et al., 2018).
38.	Trimetazidine	$C_{14}H_{22}N_2O_3$	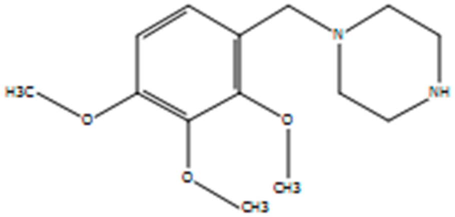	Treat angina pectoris inhibiter of long-chain 3-ketoacyl CoA thiolase activity (Kantor et al., 2000).

39.	3-Butylidene-7-hydroxyphthalide	$C_{12}H_{12}O_3$	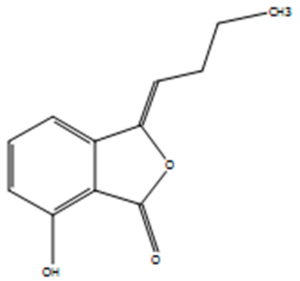	Antidiabetic activities, effective agent for the prevention or treatment of diabetes mellitus in a mammal. (D'orazio et al., 2007). Used traditionally for medicinal purposes in Asia, Europe, and North America (Leon et al., 2017). Phthalides display different biological activities including antibacterial, antifungal, insecticidal, cytotoxic, and anti-inflammatory effects (Leon et al., 2017).
40.	4-Methyldibenzothiophene	$C_{13}H_{10}S$	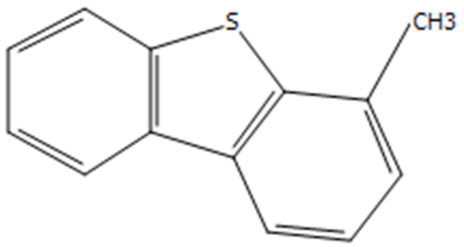	No biological activity reported.

41.	Adenine	$C_5 H_5 N_5$	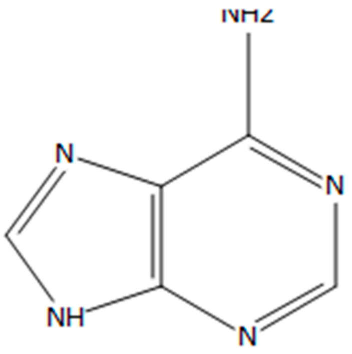	Basic unit of DNA and RNA and involved in protein synthesis (Pardee, 1954).
42.	1,2-Epoxy-3,4-butanediol 4-methanesulfonate	$C_5 H_{10} O_5 S$	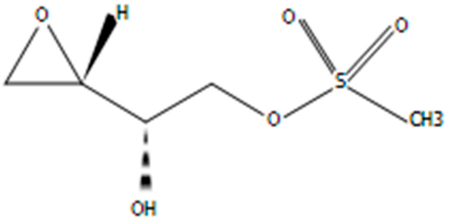	No biological activity reported.
43.	4-Methyl-3-oxoadipate	$C_7 H_{10} O_5$	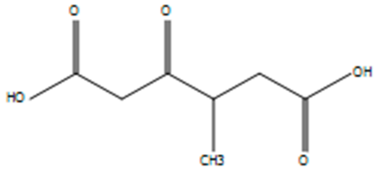	An inducer of the *mml* gene cluster in *Pseudomonas reinekei* MT1 (Marín et al., 2010). Intermediate in the degradation of 4-ML by *P. reinekei* MT1(Marín et al., 2010).
44.	Isopentenyladenine	$C_{10} H_{13} N_5$	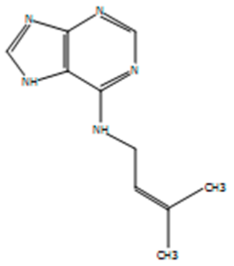	Promoting the growth of cytokinin-requiring tobacco (*Nicotiana tabacum*) callus (Laloue et al., 1977, Gajdošová et al., 2011).

				Isopentenyladenine and their ribosides exhibit anti-aging activities on skin cells and cause dedifferentiation and apoptosis in leukaemia cells (Bewley et al., 2006).
45.	Idebenone Metabolite (Benzenebutanoic acid, 2,5-dihydroxy-3,4-dimethoxy-6-methyl-)	$C_{13}H_{18}O_{6}$	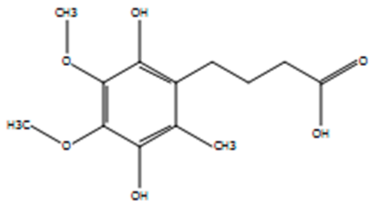	Bioactive compound isolated from the extracts of *Casuarina equisetifolia* (Pawar and Nasreen, 2018).<br><br>No biological reported activity.
46.	10-Hydroxymyristic acid methyl ester	$C_{15}H_{30}O_{3}$	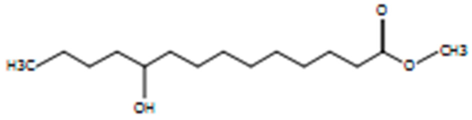	These are rare hydroxylated fatty acid methyl esters isolated from aquatic microbial sources (Littlefield-Wyer et al., 2008).
47.	(1R,2R)-3-oxo-2-pentyl-cyclopentanehexanoic acid	$C_{16}H_{28}O_{3}$	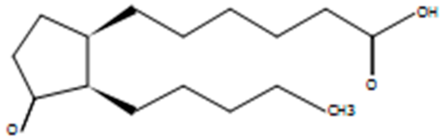	No biological reported activity.

48.	Eicosanedioic acid	$C_{20} H_{38} O_{4}$	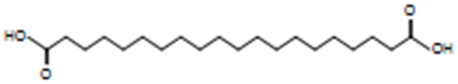	Also called arachidic acid, used for the production of detergents, photographic materials and lubricants (Shin, 2004). https://www.ebi.ac.uk/chebi/searchId.do?chebiId=CHEBI Exhibit antifungal activity (Pereira et al., 2016).
49.	Dehydrocurdione	$C_{15} H_{22} O_{2}$	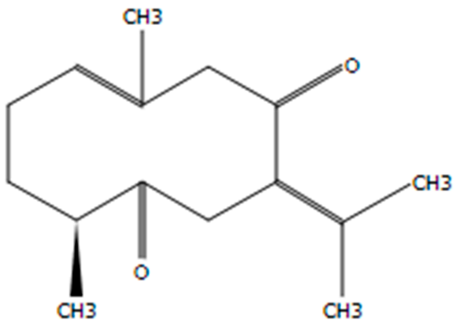	Dehydrocurdione are terpenoids show remarkable antibacterial activity against *B. subtilis* (Diastuti et al., 2014)
50.	N-Undecylbenzenesulfonic acid	$C_{17} H_{28} O_{3} S$	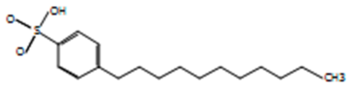	Serve as disinfectant to reduce microbial growth in foods especially in seafoods (Mixon et al., 2008).
51.	2-heptyl-nonanoic acid	$C_{16} H_{32} O_{2}$	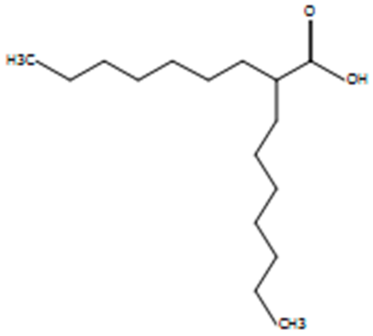	Nonanoate and its analogues exhibit board-spectrum antibacterial activities (Chakravorty et al., 2012).

52.	DMDP	$C_6H_{13}NO_4$	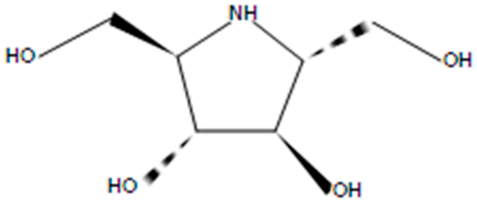	Naturally occurring sugar analogue, having activities against several plant parasitic nematode species (Birch et al., 1993).
53.	1-Monopalmitin	$C_{19}H_{38}O_4$	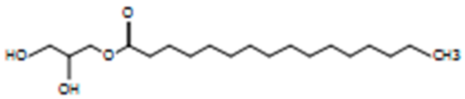	Exhibit bactericidal activities against *Helicobacter pylori* (Sun et al., 2003). No other reported activity.
54.	11,12-dihydroxy arachidic acid	$C_{20}H_{40}O_4$	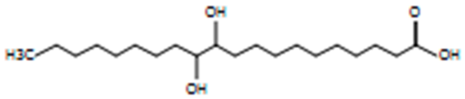	Also called arachidic acid, used for the production of detergents, photographic materials and lubricants (Shin, 2004). https://www.ebi.ac.uk/chebi/searchId.do?chebiId=CHEBI Exhibit antifungal activity (Pereira et al., 2016).

ABOURASHED, E., GALAL, A. A., M. SHEBL, A. & JABER, S. 2007. *Enhancing Effect of Isoeugenol on the Antimicrobial Activity of Isoniazid, 6-Paradol and 6-Shogaol.*

AGON, P., GOETHALS, P., VAN HAVER, D. & KAUFMAN, J. M. 1991. Permeability of the blood-brain barrier for atenolol studied by positron emission tomography. *Journal of pharmacy and pharmacology,* 43**,** 597-600.

AHGILAN, A., SABARATNAM, V. & PERIASAMY, V. 2016. Antimicrobial properties of vitamin B2. *International journal of food properties,* 19**,** 1173-1181.

AKARIM, A., PHARMA, H. & ABDEEN, M. 2015. ASSESSMENT OF PHARMACEUTICAL QUALITY CONTROL AND IN VITRO EQUIVALENCE OF VARIOUS BRANDS OF ATENOLOL (100MG) TABLETS AVAILABLE IN SUDANESE MARKET UNDER BIOWAIVER CONDITIONS.

ANDRYS, D., KULPA, D., GRZESZCZUK, M. & BIAŁECKA, B. 2018. Influence of jasmonic acid on the growth and antimicrobial and antioxidant activities of Lavandula angustifolia Mill. propagated in vitro. *Folia Horticulturae,* 30**,** 3-13.

ASANO, N., KATO, A., MIYAUCHI, M., KIZU, H., TOMIMORI, T., MATSUI, K., NASH, R. J. & MOLYNEUX, R. J. 1997. Specific α-Galactosidase Inhibitors, N-Methylcalystegines Structure/Activity Relationships of Calystegines from Lycium Chinense. *European journal of biochemistry,* 248**,** 296-303.

BAKER, B. P. & GRANT, J. A. 2018. Sodium Lauryl Sulfate Profile.

BEWLEY, J. D., BLACK, M. & HALMER, P. 2006. *The encyclopedia of seeds: science, technology and uses*, Cabi.

BIRCH, A., ROBERTSON, W., GEOGHEGAN, I., MCGAVIN, W., ALPHEY, T., PHILLIPS, M., FELLOWS, L., WATSON, A., SIMMONDS, M. & PORTER, E. 1993. DMDP-a plant-derived sugar analogue with systemic activity against plant parasitic nematodes. *Nematologica,* 39**,** 521-535.

BOBEK, M. & BLOCH, A. 1972. Synthesis and biological activity of pyrazines and pyrazine ribonucleosides as pyrimidine analogs. *Journal of Medicinal Chemistry,* 15**,** 164-168.

CAPOBIANCO, J. O., ZAKULA, D., FROST, D. J., GOLDMAN, R. C., LI, L., KLEIN, L. L. & LARTEY, P. A. 1998. Cellular Accumulation, Localization, and Activity of a Synthetic Cyclopeptamine in Fungi. *Antimicrobial Agents and Chemotherapy,* 42**,** 389-393.

CHAKRAVORTY, S., RAYNER, M. K., DE KONING, C. B., VAN VUUREN, S. F. & VAN OTTERLO, W. A. 2012. Synthesis and antimicrobial activity of the essential oil compounds (E)-and (Z)-3-hexenyl nonanoate and two analogues. *South African Journal of Chemistry,* 65**,** 202-205.

CHANG, F.-R., WEI, J.-L., TENG, C.-M. & WU, Y.-C. 1998. Two new 7-dehydroaporphine alkaloids and antiplatelet action aporphines from the leaves of Annona purpurea. *Phytochemistry,* 49**,** 2015-2018.

CHEONG, J.-J. & DO CHOI, Y. 2003. Methyl jasmonate as a vital substance in plants. *TRENDS in Genetics,* 19**,** 409-413.

COLLINI, F. J. 2000. 0.5% Polidocanol for Treatment of Varicose Veins. *Aesthetic Surgery Journal,* 20**,** 19-25.

D'ORAZIO, D., DE SAIZIEU, A., SCHUELER, G., RAEDERSTORFF, D., TEIXEIRA, S., SCHMIDT, Y. W., WEBER, P. & WOLFRAM, S. 2007. Use of phthalide derivatives for the treatment and prevention of diabetes mellitus. Google Patents.

DENG, K., LI, Q., BAI, L., GOU, Y., DONG, L., HUANG, C., WANG, S. & GAO, T. 2011. A pH/thermo-responsive injectable hydrogel system based on poly (N-acryloylglycine) as a drug carrier.

DIASTUTI, H., SYAH, Y., DEWI JULIAWATY, L. & SINGGIH, M. 2014. *Antibacterial Activity of Germacrane Type Sesquiterpenes from Curcuma heyneana Rhizomes*.

DO, Q., NGUYEN, G. T. & PHILLIPS, R. S. 2016. Inhibition of tyrosine phenol-lyase by tyrosine homologues. *Amino acids,* 48**,** 2243-2251.

DOBAK, J. D. 2017. Sodium tetradecyl sulfate formulations for treatment of adipose tissue. Google Patents.

DRABER, W. & FUJITA, T. 1992. *Rational approaches to structure, activity, and ecotoxicology of agrochemicals*, CRC Press.

EL-SEEDI, H. R., EL-BARBARY, M., EL-GHORAB, D., BOHLIN, L., BORG-KARLSON, A.-K., GORANSSON, U. & VERPOORTE, R. 2010. Recent insights into the biosynthesis and biological activities of natural xanthones. *Current medicinal chemistry,* 17**,** 854-901.

ERYILMAZ, M. & PALABIYIK, I. M. 2013. Hypochlorous acid-analytical methods and antimicrobial activity. *Tropical Journal of Pharmaceutical Research,* 12**,** 123-126.

FENG, L., MADDOX, M. M., ALAM, M. Z., TSUTSUMI, L. S., NARULA, G., BRUHN, D. F., WU, X., SANDHAUS, S., LEE, R. B., SIMMONS, C. J., TSE-DINH, Y.-C., HURDLE, J. G., LEE, R. E. & SUN, D. 2014. Synthesis, Structure–Activity Relationship Studies, and Antibacterial Evaluation of 4-Chromanones and Chalcones, as Well as Olympicin A and Derivatives. *Journal of Medicinal Chemistry,* 57**,** 8398-8420.

FERNANDES, H., SOUSA, J. P., MAIA, G. L., BARBOSA-FILHO, J. M., LIMA, E. O. & OLIVEIRA, T. L. 2013. Antibacterial activity of flavonoid 5.7. 4′-trimethoxyflavone isolated from Praxelis clematides RM King & Robinson. *Boletín Latinoamericano y del Caribe de Plantas Medicinales y Aromáticas,* 12.

GAIRE, B. P., KWON, O. W., PARK, S. H., CHUN, K.-H., KIM, S. Y., SHIN, D. Y. & CHOI, J. W. 2015. Neuroprotective effect of 6-paradol in focal cerebral ischemia involves the attenuation of neuroinflammatory responses in activated microglia. *PloS one,* 10**,** e0120203-e0120203.

GAJDOŠOVÁ, S., SPÍCHAL, L., KAMÍNEK, M., HOYEROVÁ, K., NOVÁK, O., DOBREV, P. I., GALUSZKA, P., KLÍMA, P., GAUDINOVÁ, A., ŽIŽKOVÁ, E., HANUŠ, J., DANČÁK, M., TRÁVNÍČEK, B., PEŠEK, B., KRUPIČKA, M., VAŇKOVÁ, R., STRNAD, M. & MOTYKA, V. 2011. Distribution, biological activities, metabolism, and the conceivable function of cis-zeatin-type cytokinins in plants. *Journal of Experimental Botany,* 62**,** 2827-2840.

GALAL, A. A. 2008. *Antimicrobial Activity of 6-Paradol and Related Compounds*.

GÖLCÜ, A. & YAVUZ, P. 2008. Spectral, analytical, thermal, and antimicrobial studies of novel sodium 2-[4 (2-hydroxy-3-izopropylaminopropoxy) phenyl] acetamide (atenolol) dithiocarbamate and its divalent transition metal complexes. *Russian Journal of Coordination Chemistry,* 34**,** 106-114.

GOLDMAN, M. P., KAPLAN, R. P., OKI, L. N., BENNETT, R. G. & STRICK, R. A. 1986. Extravascular effects of sclerosants in rabbit skin: a clinical and histologic examination. *The Journal of dermatologic surgery and oncology,* 12**,** 1085-1088.

GOMATHI RAJASHYAMALA, L. & ELANGO, V. 2015. Identification of bioactive components and its biological activities of Evolvulus alsinoides linn.--A GC-MS study. *Int. J. Chem. Stud,* 3**,** 41-44.

HEARD, J. T., STEINER, B. M. & TRITZ, G. J. 1981. Isolation of a catabolic product of 2-butynedioic acid that acts as a precursor of nicotinamide adenine dinucleotide. *Current Microbiology,* 5**,** 279-282.

HUDSON, T. S., CARLSON, B. A., HOENEROFF, M. J., YOUNG, H. A., SORDILLO, L., MULLER, W. J., HATFIELD, D. L. & GREEN, J. E. 2012. Selenoproteins reduce susceptibility to DMBA-induced mammary carcinogenesis. *Carcinogenesis,* 33**,** 1225-1230.

KANTOR, P. F., LUCIEN, A., KOZAK, R. & LOPASCHUK, G. D. 2000. The antianginal drug trimetazidine shifts cardiac energy metabolism from fatty acid oxidation to glucose oxidation by inhibiting mitochondrial long-chain 3-ketoacyl coenzyme A thiolase. *Circulation research,* 86**,** 580-588.

KHAN, W., PRITHIVIRAJ, B. & SMITH, D. L. 2008. Nod factor [Nod Bj V (C18:1, MeFuc)] and lumichrome enhance photosynthesis and growth of corn and soybean. *Journal of Plant Physiology,* 165**,** 1342-1351.

KUNAWAROTE, S., NAKAJIMA, M., SHIDA, K., KITASAKO, Y., FOXTON, R. M. & TAGAMI, J. 2010. Effect of dentin pretreatment with mild acidic HOCl solution on microtensile bond strength and surface pH. *Journal of dentistry,* 38**,** 261-268.

LALOUE, M., TERRINE, C. & GUERN, J. 1977. Cytokinins: Metabolism and Biological Activity of N-(Delta-Isopentenyl)adenosine and N-(Delta-Isopentenyl)adenine in Tobacco Cells and Callus. *Plant physiology,* 59**,** 478-483.

LEAL, J. S., GONZALEZ, J., COMELLES, F., CAMPOS, E. & CIGANDA, T. 1991. Biodegradability and toxicity of anionic surfactants. *Acta hydrochimica et hydrobiologica,* 19**,** 703-709.

LEE, M.-R., RAMAN, N., GELLMAN, S. H., LYNN, D. M. & PALECEK, S. P. 2014. Hydrophobicity and Helicity Regulate the Antifungal Activity of 14-Helical β-Peptides. *ACS Chemical Biology,* 9**,** 1613-1621.

LEON, A., DEL-ANGEL, M., AVILA, J. L. & DELGADO, G. 2017. Phthalides: Distribution in Nature, Chemical Reactivity, Synthesis, and Biological Activity. *Prog Chem Org Nat Prod,* 104**,** 127-246.

LITTLEFIELD-WYER, J., BROOKS, P. & KATOULI, M. 2008. Application of biochemical fingerprinting and fatty acid methyl ester profiling to assess the effect of the pesticide Atradex on aquatic microbial communities. *Environmental pollution,* 153**,** 393-400.

LÓPEZ-MUÑOZ, F. & ALAMO, C. 2013. Active metabolites as antidepressant drugs: the role of norquetiapine in the mechanism of action of quetiapine in the treatment of mood disorders. *Frontiers in psychiatry,* 4**,** 102-102.

MARÍN, M., PÉREZ-PANTOJA, D., DONOSO, R., WRAY, V., GONZÁLEZ, B. & PIEPER, D. H. 2010. Modified 3-Oxoadipate Pathway for the Biodegradation of Methylaromatics in Pseudomonas reinekei MT1. *Journal of Bacteriology,* 192**,** 1543-1552.

MASSARO, C. F., SHELLEY, D., HEARD, T. A. & BROOKS, P. 2014. In Vitro Antibacterial Phenolic Extracts from "Sugarbag" Pot-Honeys of Australian Stingless Bees (Tetragonula carbonaria). *Journal of Agricultural and Food Chemistry,* 62**,** 12209-12217.

MATOLCSY, G., NÁDASY, M. & ANDRISKA, V. 1989. *Pesticide chemistry,* Elsevier.

MCKENNA, S. M. & DAVIES, K. J. 1988. The inhibition of bacterial growth by hypochlorous acid. Possible role in the bactericidal activity of phagocytes. *The Biochemical journal,* 254**,** 685-692.

MEIJA, J. & CARUSO, J. A. 2004. Selenium and sulfur trichalcogenides from the chalcogenide exchange reaction. *Inorganic chemistry,* 43**,** 7486-7492.

MIMANNE, G., SENNOUR, R., BENGHALEM, A., TALEB, S. & BENHABIB, K. 2012. Use of the Geomaterial for the Elimination of Surfactant Dodecylbenzene Sulfonic Acid from Aqueous Solutions. *Journal of Material environment science,* 3**,** 712-725.

MIXON, S. P., SMITHYMAN, D. M. & DAUTREUIL, F. 2008. Methods for reducing microbial contamination in seafood processing. Google Patents.

MOON, S., HWANG, E. & CHO, K. 2005. Treatment of pyogenic granuloma by sodium tetradecyl sulfate sclerotherapy. *Archives of Dermatology,* 141**,** 644-646.

OPDYKE, D. 1979. Monographs on fragrance raw materials. *Food and cosmetics toxicology,* 17**,** 509-533.

PALLELA, R. & EHRLICH, H. 2016. *Marine Sponges: Chemicobiological and Biomedical Applications*, Springer.

PARDEE, A. B. 1954. Nucleic acid precursors and protein synthesis. *Proceedings of the National Academy of Sciences of the United States of America,* 40**,** 263.

PATTISON, D. I. & DAVIES, M. J. 2001. Absolute rate constants for the reaction of hypochlorous acid with protein side chains and peptide bonds. *Chemical research in toxicology,* 14**,** 1453-1464.

PAWAR, B., SHINDE, V. & CHASKAR, A. 2013. n-Dodecylbenzene Sulfonic Acid (DBSA) as a Novel Brønsted Acid Catalyst for the Synthesis of Bis (indolyl) methanes and Bis (4-hydroxycoumarin-3-yl) methanes in Water. *Green and Sustainable Chemistry,* 3**,** 56.

PAWAR, D. S. & NASREEN, S. 2018. HR-LCMS of phytoconstituents and antifungal activity of medicinal plants. *Journal of Medicinal Plants,* 6**,** 173-176.

PEREIRA, C. B., PEREIRA DE SA, N., BORELLI, B. M., ROSA, C. A., BARBEIRA, P. J. S., COTA, B. B. & JOHANN, S. 2016. Antifungal activity of eicosanoic acids isolated from the endophytic fungus Mycosphaerella sp. against Cryptococcus neoformans and C. gattii. *Microb Pathog,* 100**,** 205-212.

PIPER, W. & MAXWELL, K. 1971. Mode of action of surfactants on mosquito pupae. *Journal of economic entomology,* 64**,** 601-606.

RAUHA, J.-P. 2001. The search for biological activity in Finnish plant extracts containing phenolic compounds.

ROTZINGER, S., BOURIN, M., AKIMOTO, Y., COUTTS, R. T. & BAKER, G. B. 1999. Metabolism of some "second"- and "fourth"-generation antidepressants: iprindole, viloxazine, bupropion, mianserin, maprotiline, trazodone, nefazodone, and venlafaxine. *Cell Mol Neurobiol,* 19**,** 427-42.

SADICK, N. S., SENTERFIT, L. B. & KLEIN, R. F. 1996. The intrinsic antimicrobial activity of selected sclerosing agents in sclerotherapy. *Dermatol Surg,* 22**,** 369-71.

SAINIS, I., BANTI, C. N., OWCZARZAK, A. M., KYROS, L., KOURKOUMELIS, N., KUBICKI, M. & HADJIKAKOU, S. K. 2016. New antibacterial, non-genotoxic materials, derived

from the functionalization of the anti-thyroid drug methimazole with silver ions. *Journal of Inorganic Biochemistry,* 160, 114-124.

SÁNCHEZ-PATÁN, F., CHIOUA, M., GARRIDO, I., CUEVA, C., SAMADI, A., MARCO-CONTELLES, J., MORENO-ARRIBAS, M. V., BARTOLOMÉ, B. & MONAGAS, M. 2011. Synthesis, analytical features, and biological relevance of 5-(3′, 4′-dihydroxyphenyl)-γ-valerolactone, a microbial metabolite derived from the catabolism of dietary flavan-3-ols. *Journal of agricultural and food chemistry,* 59, 7083-7091.

SCHWEIGER, D., BAUFELD, C., DRESCHER, P., OLTROGGE, B., HÖPFNER, S., MESS, A., LÜTTKE, J., RIPPKE, F., FILBRY, A. & MAX, H. 2013. Efficacy of a New Tonic Containing Urea, Lactate, Polidocanol, and <i>Glycyrrhiza inflata</i> Root Extract in the Treatment of a Dry, Itchy, and Subclinically Inflamed Scalp. *Skin Pharmacology and Physiology,* 26, 108-118.

SHI, M., SEKULOVSKI, N., MACLEAN, J. A., 2ND & HAYASHI, K. 2018. Prenatal Exposure to Bisphenol A Analogues on Male Reproductive Functions in Mice. *Toxicol Sci,* 163, 620-631.

SHIN, H. 2004. Lipid composition and nutritional and physiological roles of perilla seed and its oil. *Perilla, The Genus Perilla. Taylor & Francis, London*, 93-108.

SINGER, M. M. & TJEERDEMA, R. S. 1993. Fate and effects of the surfactant sodium dodecyl sulfate. *Reviews of environmental contamination and toxicology.* Springer.

SPEIDEL, J. T., XU, M. & ABDEL-RAHMAN, S. Z. 2018. Bisphenol A (BPA) and bisphenol S (BPS) alter the promoter activity of the ABCB1 gene encoding P-glycoprotein in the human placenta in a haplotype-dependent manner. *Toxicol Appl Pharmacol,* 359, 47-54.

STEVENS, J. F., TAYLOR, A. W., CLAWSON, J. E. & DEINZER, M. L. 1999. Fate of xanthohumol and related prenylflavonoids from hops to beer. *J Agric Food Chem,* 47, 2421-8.

SUN, H., WANG, D., SONG, X., ZHANG, Y., DING, W., PENG, X., ZHANG, X., LI, Y., MA, Y. & WANG, R. 2017. Natural prenylchalconaringenins and prenylnaringenins as antidiabetic agents: α-glucosidase and α-amylase inhibition and in vivo antihyperglycemic and antihyperlipidemic effects. *Journal of agricultural and food chemistry,* 65, 1574-1581.

SÜZGEÇ-SELÇUK, S. & BIRTEKSÖZ, A. 2011. Flavonoids of Helichrysum chasmolycicum and its antioxidant and antimicrobial activities. *South African Journal of Botany,* 77, 170-174.

TADA, H. & YASUDA, F. 1984. Siphonodiol, a new polyacetylenic metabolite from the sponge Siphonochalina truncate. *Chemistry Letters,* 13, 779-780.

TIAN, F., JIA, Y., ZHANG, Y., SONG, W., ZHAO, G., QU, Z., LI, C., CHEN, Y. & LI, P. 2016. A HClO-specific near-infrared fluorescent probe for determination of Myeloperoxidase activity and imaging mitochondrial HClO in living cells. *Biosens Bioelectron,* 86, 68-74.

URQUIZA, N., ISLAS, M., LAURA DITTLER, M., MOYANO, M. A., MANCA, S., LEZAMA, L., ROJO, T., MARTÍNEZ, J., DIEZ, M., TÉVEZ, L., A.M. WILLIAMS, P. & FERRER, E. 2013. *Inhibition behavior on alkaline phosphatase activity, antibacterial and antioxidant activities of ternary methimazole-phenanthroline-copper(II) complex*.

WANG, L., BASSIRI, M., NAJAFI, R., NAJAFI, K., YANG, J., KHOSROVI, B., HWONG, W., BARATI, E., BELISLE, B. & CELERI, C. 2007. Hypochlorous acid as a potential wound

care agent: part I. Stabilized hypochlorous acid: a component of the inorganic armamentarium of innate immunity. *Journal of burns and wounds,* 6.

WEIQING, H. Z. Z., ZONGDE, J. Z. Q. W. W. & JINZHU, C. 2009. Repellent Activity of Terpenoids against Tribolium castanenum (Herbst) Adults. *Journal of the Chinese Cereals and Oils Association,* 12**,** 025.

WU, S.-J., CHEN, I.-S., CHEM, C.-Y., TENG, C.-M. & WU, T.-S. 1996. Structure and Synthesis of Simulansamide, a Platelet Aggregation Inhibitor from Zanthoxylum Simulans. *Journal of the Chinese Chemical Society,* 43**,** 195-198.

YANNAI, S. 2003. *Dictionary of food compounds with CD-ROM: Additives, flavors, and ingredients*, Chapman and Hall/CRC.

ZAHER, A. M., MOHARRAM, A. M., DAVIS, R., PANIZZI, P., MAKBOUL, M. A. & CALDERÓN, A. I. 2015. Characterisation of the metabolites of an antibacterial endophyte Botryodiplodia theobromae Pat. of Dracaena draco L. by LC–MS/MS. *Natural product research,* 29**,** 2275-2281.

ZHOU, J., XIE, G. & YAN, X. 2011. Encyclopedia of traditional Chinese medicines. *Isolat Compound AB,* 1**,** 455.
